# PICK1 Deficiency Impairs Secretory Vesicle Biogenesis and Leads to Growth Retardation and Decreased Glucose Tolerance

**DOI:** 10.1371/journal.pbio.1001542

**Published:** 2013-04-23

**Authors:** Birgitte Holst, Kenneth L. Madsen, Anna M. Jansen, Chunyu Jin, Mattias Rickhag, Viktor K. Lund, Morten Jensen, Vikram Bhatia, Gunnar Sørensen, Andreas N. Madsen, Zhichao Xue, Siri K. Møller, David Woldbye, Klaus Qvortrup, Richard Huganir, Dimitrios Stamou, Ole Kjærulff, Ulrik Gether

**Affiliations:** 1 Laboratory for Molecular Pharmacology, Novo Nordisk Foundation Center for Basic Metabolic Research, The Faculty of Health Sciences, Panum Institute, University of Copenhagen, Copenhagen, Denmark; 2 Department of Neuroscience and Pharmacology, The Faculty of Health Sciences, Panum Institute, University of Copenhagen, Copenhagen, Denmark; 3 Molecular Neuropharmacology Laboratory, The Faculty of Health Sciences, Panum Institute, University of Copenhagen, Copenhagen, Denmark; 4 BioNano Laboratory, The Faculty of Health Sciences, Panum Institute, University of Copenhagen, Copenhagen, Denmark; 5 Core Facility for Integrated Microscopy, Department of Biomedical Science, Faculty of Health Sciences, University of Copenhagen, Copenhagen, Denmark; 6 Department of Neuroscience, The Howard Hughes Medical Institute, The Johns Hopkins University School of Medicine, Baltimore, Maryland, United States of America; University of Cambridge, United Kingdom

## Abstract

Secretory vesicles in endocrine cells store hormones such as growth hormone (GH) and insulin before their release into the bloodstream. The molecular mechanisms governing budding of immature secretory vesicles from the trans-Golgi network (TGN) and their subsequent maturation remain unclear. Here, we identify the lipid binding BAR (Bin/amphiphysin/Rvs) domain protein PICK1 (protein interacting with C kinase 1) as a key component early in the biogenesis of secretory vesicles in GH-producing cells. Both PICK1-deficient *Drosophila* and mice displayed somatic growth retardation. Growth retardation was rescued in flies by reintroducing PICK1 in neurosecretory cells producing somatotropic peptides. PICK1-deficient mice were characterized by decreased body weight and length, increased fat accumulation, impaired GH secretion, and decreased storage of GH in the pituitary. Decreased GH storage was supported by electron microscopy showing prominent reduction in secretory vesicle number. Evidence was also obtained for impaired insulin secretion associated with decreased glucose tolerance. PICK1 localized in cells to immature secretory vesicles, and the PICK1 BAR domain was shown by live imaging to associate with vesicles budding from the TGN and to possess membrane-sculpting properties in vitro. In mouse pituitary, PICK1 co-localized with the BAR domain protein ICA69, and PICK1 deficiency abolished ICA69 protein expression. In the *Drosophila* brain, PICK1 and ICA69 co-immunoprecipitated and showed mutually dependent expression. Finally, both in a *Drosophila* model of type 2 diabetes and in high-fat-diet-induced obese mice, we observed up-regulation of PICK1 mRNA expression. Our findings suggest that PICK1, together with ICA69, is critical during budding of immature secretory vesicles from the TGN and thus for vesicular storage of GH and possibly other hormones. The data link two BAR domain proteins to membrane remodeling processes in the secretory pathway of peptidergic endocrine cells and support an important role of PICK1/ICA69 in maintenance of metabolic homeostasis.

## Abstract 2

Two lipid membrane sculpting BAR domain proteins, PICK1 and ICA69, play a key role early in the biogenesis of peptide hormone secretory vesicles and are critical for normal growth and metabolic homeostasis.

## Author Summary

Regulated secretion of peptide hormones, such as growth hormone (GH) and insulin, represents a fundamental process in controlling physiological homeostasis. In endocrine cells, hormone-containing vesicles bud from the Golgi apparatus to enable storage and regulated release into the blood stream. Here we show that two proteins with a lipid membrane-shaping BAR domain, PICK1 and ICA69, work together in the pituitary gland and the pancreas to facilitate the budding of early secretory vesicle from the Golgi apparatus. The physiological significance of our findings was borne out by showing that mice and *Drosophila* flies lacking the PICK1 encoding gene have marked growth retardation. PICK1-deficient mice showed increased fat accumulation, reduced body weight and length, as well as reduced glucose clearance from the blood stream. Consistent with these findings, we observed a severe reduction in GH storage in the pituitary and impaired secretion of both insulin and GH in response to physiological stimuli. Finally, we found that PICK1 expression levels were raised in a fly model of type 2 diabetes and in high-fat-diet-induced obese mice. These results indicate that alteration of PICK1 expression might play a role in pathophysiological processes of metabolic diseases and/or in a protective compensatory mechanism.

## Introduction

The regulated secretory pathway is a hallmark of endocrine, exocrine, and neuronal cells and involves formation of secretory vesicles to enable storage and regulated secretion of cargo molecules [1],[2]. Peptide hormones and neuropeptides are synthesized as larger precursors in the endoplasmic reticulum and targeted to the Golgi apparatus. At the trans-Golgi network, the precursors are packaged into immature secretory vesicles that bud from the TGN. During maturation of the immature vesicles, the precursors are processed to active hormones, which are secreted in a regulated manner by fusion of the secretory vesicles with the plasma membrane. Although several steps in the regulated secretory pathway have been characterized, the mechanism underlying biogenesis of the secretory vesicles is still poorly understood [1],[2]. A key question is, which molecular components that are responsible for the membrane remodeling and reshaping that is required for budding of vesicles from the TGN and the subsequent maturation of these vesicles. Previous studies indicated that generation of the necessary membrane curvature at the TGN depends on lipids such as diacylglycerol, phosphatidic acid, and cholesterol [1],[3]–[5]. In addition, it has been suggested that hormone precursors aggregate together with chromogranins in membrane rafts of the TGN, thereby providing a driving force for vesicle budding [1],[6],[7]. The general importance of chromogranins has been substantiated both by depletion experiments in endocrine cells and overexpression experiments in non-endocrine fibroblast-like cells leading to “de novo” formation of dense core-like granules containing chromogranin [1],[6],[7]. However, the exact molecular components involved in secretory vesicle formation remain unknown.

BAR (Bin/amphiphysin/Rvs) domains are crescent-shaped dimeric α-helical modules that are essential in dynamic remodeling of lipid membranes by means of their unique capacity to both sense and impose membrane curvature [8]–[11]. BAR-domain proteins have been implicated in a variety of cellular functions including clathrin-mediated endocytosis where at least four different BAR-domain proteins, including FCHO1/2, endophilin, amphiphysin, and SNX9, are known to be involved in the vesicle budding and fission process [12],[13]. Analogous to the plasma membrane, the TGN is a “hotspot” for membrane remodeling and vesicle formation; however, the processes occurring at the TGN have not been characterized in similar details.

Protein interacting with C kinase 1 (PICK1) is a BAR domain protein with homology to the BAR domain proteins ICA69 (islet cell autoantigen 69) and arfaptin 1/2 [14]–[18]. The protein is widely distributed in the body with highest known expression in the brain, pancreas, and testis [19]. In the brain, PICK1 is found in many different regions [19] and known for regulating trafficking and phosphorylation of neurotransmitter receptors, such as the α-amino-3-hydroxy-5-methyl-4-isoxazolepropionic acid (AMPA)-type ionotropic glutamate receptor, to which PICK1 binds via an N-terminal PSD-95/Discs-large/ZO-1 homology (PDZ) domain [14]–[17]. Recently, we unexpectedly discovered that in the *Drosophila* brain, PICK1 is almost exclusively expressed in peptidergic neurons [20]. Peptide hormones released from these neurosecretory cells are critical for growth and metabolism, but the functional significance of PICK1 expression in these cells is unknown. Of further interest, previous data have suggested that PICK1 exists in part in a heterodimeric complex with the homologous BAR domain protein, ICA69 [19]. ICA69 was originally identified as diabetes-associated autoantigen in islet cells of the pancreas and is functionally poorly characterized [21]. It contains an N-terminal BAR domain with homology to the PICK1 BAR domain and a rather long C-terminal domain of unknown structure and function [19]. ICA69 has been suggested to regulate neurosecretory processes [22] and to prevent synaptic targeting/localization of PICK1 [19].

Here, we show that PICK1 is not only expressed in neurosecretory cells of *Drosophila* but also in hormone-producing cells of the mouse pituitary. Phenotypic characterization of mutant PICK1-deficient flies and mice reveal that both are characterized by marked somatic growth retardation. In the flies, this can be rescued by reintroducing PICK1 selectively in peptidergic neurosecretory cells. In the PICK1-deficient mice, we find clear signs of GH deficiency that can be attributed to reduced formation of GH-containing dense core secretory vesicles in the pituitary. We find moreover evidence for decreased levels of prolactin in the pituitary as well as for impaired insulin secretion from the pancreas. Our further analyses indicate a transient localization of PICK1 to early secretory vesicles budding from the TGN, and in vitro we find that the PICK1 BAR-domain can actively sculpt lipid membranes. We also obtain evidence that PICK1 exerts its action in endocrine cells in a heterodimeric complex with ICA69. A more general role of the PICK1/ICA69 complex in secretory vesicle formation is supported by the observation that PICK1 and ICA69 promote vesicular chromogranin localization in non-endocrine COS7 cells. Furthermore, PICK1 mRNA expression is up-regulated in both a *Drosophila* model of type 2 diabetes (T2D) and in high-fat-diet-induced obese mice. Summarized, our results suggest a hitherto unknown key role of PICK1 and ICA69 in secretory vesicle formation at the TGN and indicate a putative role of PICK1 during metabolic disease.

## Results

### Growth Retardation in PICK1-Null *Drosophila*

**Figure 1:**
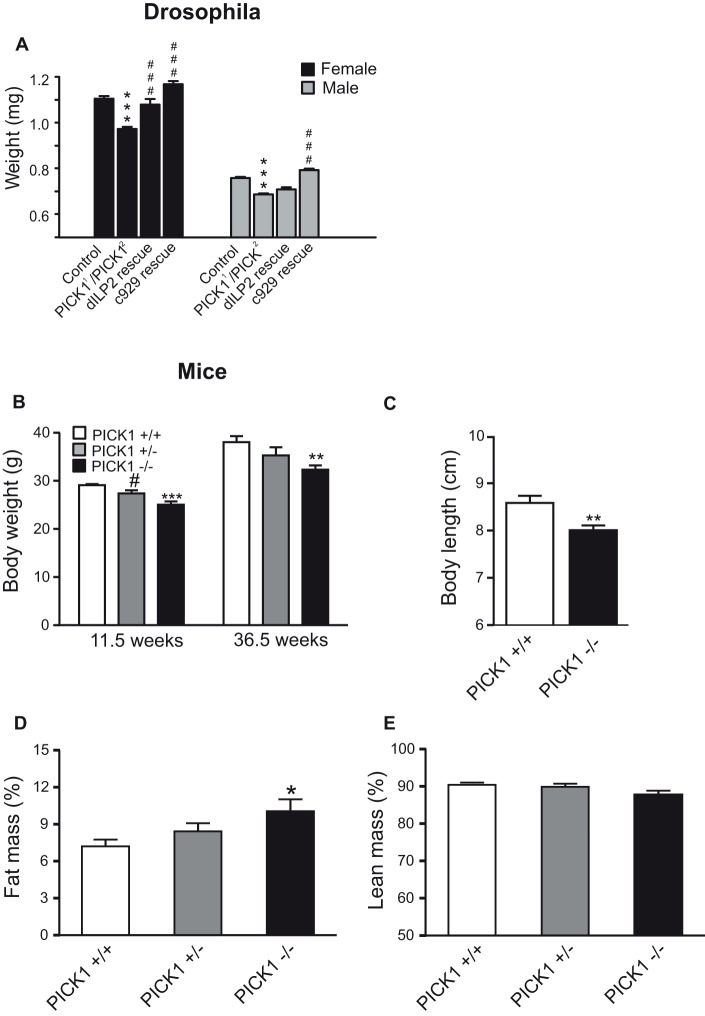
Involvement of PICK1 in somatic growth and body composition. (A) Body weight of female (black) and male (grey) control *Drosophila* flies, *Drosophila PICK1*-null mutants, and rescued mutants (dILP and c929). Rescued mutants included *PICK1^1^*/*PICK^2^* transheterozygotes with expression of HA-tagged *UAS-PICK1* transgene either under the dILP2 promotor to drive expression in peptidergic cells secreting *Drosophila* insulin-like peptides (dILPs) [24], or under the generally peptidergic *DIMM* promoter (c929) [20],[23]. Data are means ± SE of at least 15 data points, where each point represents the average from weighing 3–8 flies. Genotypes: “*c929* rescue,” *c929-GAL4 PICK^1^/PICK^2^ UAS-PICK1-HA*; “*dILP2* rescue,” *PICK^1^/PICK^2^ UAS-PICK-HA; dILP2/+*. ***p<0.0001 (compared to Control); ^###^ *p*<0.0001 (compared to *PICK^1^/PICK^2^*). (B) PICK1-deficient mice display reduced body weight at both age ∼11.5 wk and age 36.5 wk. Data are means ± SE (*n*≥7). **p<0.01, ***p<0.001, compared to WT mice. ^#^ *p*<0.05, compared to PICK1-deficient mice. (C) PICK1-deficient mice display reduced body length between nose and tail root. Measurements were performed in anesthetized mice at 10–12 wk of age. Data are means ± SE (*n* = 11). *p<0.01, compared to WT mice. (D and E) PICK1-deficient mice display altered body composition. Fat mass and lean mass were determined in unanesthetized mice at 15–17 wk of age by quantitative magnetic resonance imaging. Data are presented as percent of total body weight (means ± SE, *n* = 7–10). *p<0.05, compared to WT mice.

By immunostaining we observed an unanticipated PICK1 signal in neurosecretory cells of *Drosophila melanogaster* [20]. Because some of these cells secrete peptides governing growth and metabolism, we compared the body weight of *Drosophila* PICK1-null mutants with wild-type (WT) flies. Adult flies, which were homozygous or transheterozygous for two *PICK1*-null alleles, *PICK1^1^* and *PICK1^2^* [20], exhibited significant weight loss compared to controls (Figure 1A). The weight loss was fully rescued in both females and males when expressing PICK1 under control of the *DIMM* promoter, using the *c929-GAL4* driver line (Figure 1A) [20],[23]. This is in agreement with *DIMM* encoding a transcription factor important for differentiation of peptidergic cells and with the observation that the expression of PICK1 and DIMM largely overlaps [20],[23]. In *Drosophila*, somatic growth and energy homeostasis is influenced by a family of insulin-like peptides (dILPs) that function equivalently to growth hormone (GH) and insulin in mammals [24]. When driving PICK1 expression under the dILP2 promoter, normal growth was rescued in females with a nonsignificant trend in males (Figure 1A). Together, the results suggest that to ensure proper growth regulation in *Drosophila*, PICK1 must be expressed in peptidergic cells, of which cells secreting dILPs play a major role.

### Somatic Growth Retardation and GH Deficiency in PICK1-Deficient Mice

To probe the importance of PICK1 for neuroendocrine function in mammals, we studied mice with targeted disruption of the PICK1 gene (PICK1-deficient mice) [14]. In accordance with our *Drosophila* findings, PICK1-deficient mice displayed significantly lower body weight and length compared to WT littermates (Figure 1B–C). To evaluate the body composition of the mice, we performed MRI scans, which revealed significantly higher fat percentage in PICK1-deficient animals (Figure 1D). In contrast, the percentage of lean body mass was unchanged (Figure 1E). However, as expected from the lower body weight and length, the absolute lean mass in grams was significantly decreased in PICK1-deficient mice (Figure S1A–B).

**Figure 2:**
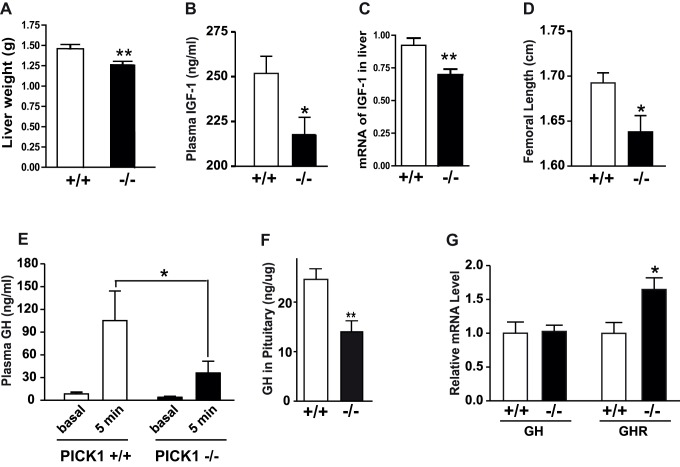
Decreased GH storage and secretion in PICK1-deficient mice. (A) The livers of PICK-1-deficient mice had a significantly lower weight than those of WT mice (**p<0.01). (B) PICK1-deficient mice exhibited a decreased level of plasma IGF-1 (*p<0.05). (C) Decreased relative expression level of IGF-1 mRNA in the liver of PICK1-deficient mice versus WT mice (**p = 0.0045). Measurements in (A), (B), and (C) were performed in samples from 35–38-wk-old WT (+/+) or PICK1-deficient mice (−/−) after overnight fast. Data are means ± SE (*n* = 10–14). (D) PICK1-deficient mice display reduced femoral length. Measurements were done on 39–41-wk-old WT (+/+) and PICK1-deficient mice (−/−). Data are means ± SE (*n* = 4–5). *p<0.05, compared to WT. (E) PICK1-deficient mice display decreased ghrelin-induced GH secretion. Basal plasma GH level and the level 5 min after ghrelin administration were determined in anesthetized WT (+/+) and PICK1-deficient mice (−/−) at the age of 7–9 wk. White bars, WT (+/+); black bars, PICK1-deficient mice (−/−). Data are means ± SE (*n* = 7–9). *p<0.05, compared to WT. (F) PICK1-deficient mice display decreased GH content in the pituitary. Pituitaries were taken from 34-wk-old WT (+/+) or PICK1-deficient mice (−/−). Data are means ± SE (*n* = 4). **p<0.01. (G) Relative expression levels of GH and GHR mRNA in pituitaries, determined by RT-PCR analysis (35–38-wk-old mice). White bars, PICK1 +/+: black bars, PICK1 −/−. Data are means ± SE (*n* = 7–8). *p<0.05.

The PICK1-deficient mice did not differ significantly from WT littermates in their food and water intake, energy expenditure, or preference for fuel consumption, as determined in calorimetric cages over 72 h (Figure S1C–H). This suggested that the growth impairment and change in body composition were likely the result of a defect in the function of anabolic hormones. Surmising that GH might be a prime candidate, we measured liver weight, IGF-1 (insulin-like growth factor-1) levels, and femur length, all well-established markers of the average daily GH secretion [25]. Note that because GH is secreted in a pulsatile manner, direct measurements of plasma GH do not reliably assess total GH secretion. Indeed, the weight of the liver, the femur length, and both the IGF-1 plasma level and liver mRNA level were significantly decreased in PICK1-deficient mice (Figure 2A–D). To assess the ability of the mice to secrete GH in response to a physiologically relevant stimulus, we measured the plasma level of GH 5 min after treatment with ghrelin [26]. In PICK1-deficient mice, the plasma levels of GH were significantly lower after ghrelin than in littermate controls (Figure 2E). This decrease in secretion might be the result of impaired hormone storage since the total content of GH in the pituitary was markedly decreased in PICK1-deficient mice, as measured by ELISA (Figure 2F). The content of prolactin was also significantly decreased, whereas a tendency toward reduction was seen for ACTH and TSH (Figure S2). The impaired storage of GH was not caused by decreased transcriptional activity, because quantitative PCR revealed similar GH mRNA levels in WT and PICK1-deficient mice (Figure 2G). In contrast, the GH receptor mRNA levels were up-regulated (Figure 2G), possibly reflecting a mechanism to compensate for the decreased GH level.

### Impaired Glucose Tolerance and Reduced Insulin Secretion in PICK1-Deficient Mice

**Figure 3:**
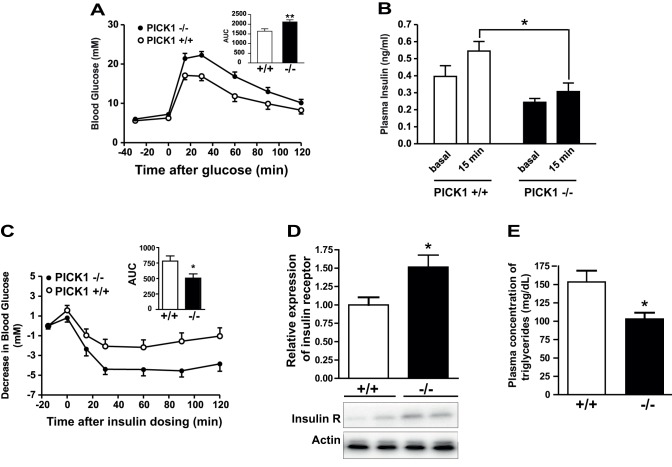
Dysregulation of glucose homeostasis in PICK1-deficient mice. (A) Impaired glucose tolerance in PICK1-deficient mice. OGTT was done in 10–13-wk-old WT (+/+) and PICK1-deficient mice (−/−) after overnight fast. White circles, PICK1 +/+; black circles, PICK1 −/−. The inset shows area under curve (AUC). Data are means ± SE (*n* = 9). **p<0.01. (B) Mice deficient in PICK1 display decreased insulin secretion during OGTT. White bars, PICK1 +/+: black bars, PICK1 −/−. Data are means ± SE. *p<0.05. (C) Mice deficient in PICK1 (27–29 wk) display preserved or even improved insulin sensitivity in the ITT. White circles, PICK1 +/+; black circles, PICK1 −/−. Data are means ± SE (*n* = 9–11). AUC was significantly lower for the PICK1-deficient mice compared to WT mice. *p<0.05. (D) The protein expression level of the insulin receptor as determined by quantification of Western blots on liver samples from 35–38-wk-old mice after overnight fasting was significantly increased in PICK1-deficient mice (−/−) compared to WT (+/+) mice (means ± SE, *p = 0.024). Western blots with insulin receptor and actin immunoreactivity of two representative samples out of 13 PICK−/− and 10 PICK+/+ samples are shown below. (E) The plasma levels of triglycerides (TG) after overnight fasting were significantly decreased in PICK1-deficient mice (−/−) compared to WT (+/+) mice (154±15 versus 103±9, *n* = 10–13, **p = 0.0056).

To characterize further the influence of PICK1 on metabolism, we performed oral glucose tolerance tests (OGTTs). In response to the glucose load, we observed a significant delay in plasma glucose clearance (Figure 3A) and, consistent with this, also a decrease in insulin secretion in PICK1-deficient mice (Figure 3B). These findings indicate that not only GH secretion but possibly also insulin secretion is impaired in the absence of PICK1. The impaired glucose tolerance was not due to the insulin resistance commonly observed in obese animal models of T2D. On the contrary, an insulin tolerance test (ITT) revealed increased insulin sensitivity in the PICK1-deficient mice as illustrated by a significant reduction in AUC (area under curve) (Figure 3C). In accordance with the increased insulin sensitivity, we observed increased insulin receptor expression in the liver (Figure 3D). Interestingly, increased insulin sensitivity has previously been observed in animal models characterized by impaired GH action [27]. Finally, we found decreased plasma triglyceride levels (Figure 3E), as described in other rodent models of GH deficiency [28]. Together, our data suggest that the metabolic phenotype of PICK1-deficient mice is characterized to a large degree by a GH deficit. In addition, the results suggest an impairment of insulin secretion in agreement with work by Xia and colleagues [29].

### Rescue of the Metabolic Phenotype Observed in PICK1-Deficient Mice by Exogenous Administration of GH

**Figure 4:**
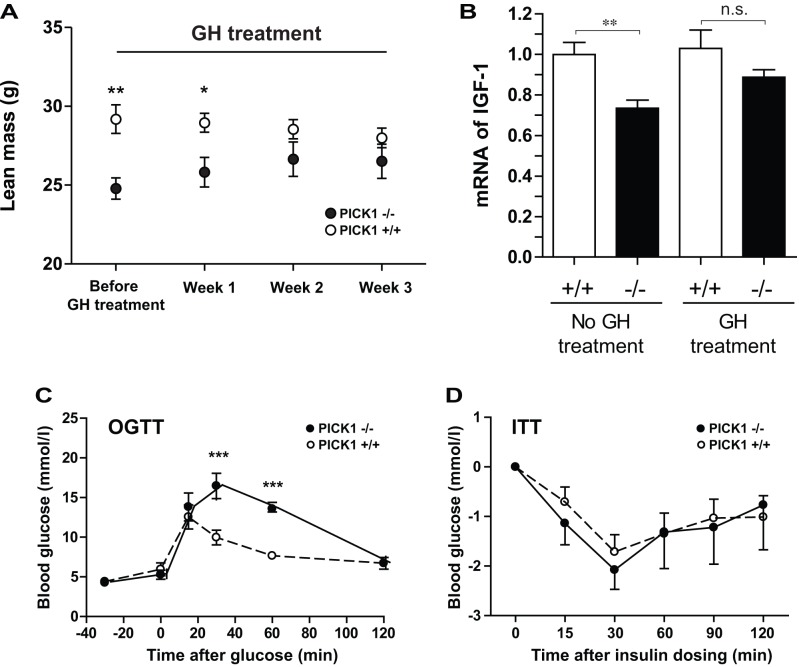
Rescue of the phenotype of PICK1-deficient mice by GH administration. (A) Lean body mass was evaluated by MRI scanning before GH administration and subsequently once every week both in PICK1-deficient mice (black squares) and in saline-treated WT littermates (white circles). Before treatment the difference between the two groups was highly significant (**p = 0.0027), after a week the difference decreased but was still significant (*p = 0.050), and after 2 and 3 wk no significant difference were observed. (B) IGF-1 mRNA level in the liver as determined by RT-PCR was significantly lower in PICK1-deficient mice compared to untreated mice (*p = 0.0045), however this difference was not observed after GH-treatment for 3 wk. (C) OGTT and ITT on GH-treated PICK1-deficient mice (black circles) and the saline-treated WT littermate controls (white circles). Significant difference was observed in the OGTT, whereas no difference was observed in ITT. Data are expressed as mean ± SE and analyzed by two-way ANOVA in (A) and Student's *t* test in (B–D) (*n* = 4–8 in all the experiments). All experiments have been reproduced in another cohort of mice (*n* = 3–4), where the age of the mice was 4 wk higher.

To elucidate better the contribution of the GH deficiency to the phenotype observed in the PICK1-deficient mice, we administrated GH once daily to adult (12–16 wk old) mice for 3 wk. Before the treatment was initiated, we measured body weight and body composition of the mice as well as performed oral glucose tolerance and ITTs. Importantly, the data described in Figures 2 and 3 were confirmed in this cohort of mice (unpublished data). During the treatment period, the body composition was measured every week. The PICK1-deficient mice gradually approached the lean body mass of the littermate WT control mice and were indistinguishable from these already after 2 wk of treatment (Figure 4A), consistent with a rescue of the phenotype by GH administration. We also observed a rescue of the strongly decreased IGF-1 mRNA levels in the liver of the PICK1-deficient mice—that is, the IGF-1 mRNA level in the liver after GH treatment was not significantly different from WT littermate control mice (Figure 4B). The GH treatment, however, did not affect glucose tolerance (Figure 4C). In contrast, the increased insulin sensitivity was rescued by GH administration, as no difference between PICK1-deficient mice treated with GH and wild-type littermate controls was observed (Figure 3C versus Figure 4D). In summary, the data substantiate the contribution of the GH deficiency to the metabolic phenotype of PICK1-deficent mice and emphasize the conceivable additional contribution from impaired insulin secretion.

### PICK1 Is Expressed in the Pituitary and PICK1-Deficiency Leads to a Reduced Pool of Dense Core Secretory Vesicles

**Figure 5:**
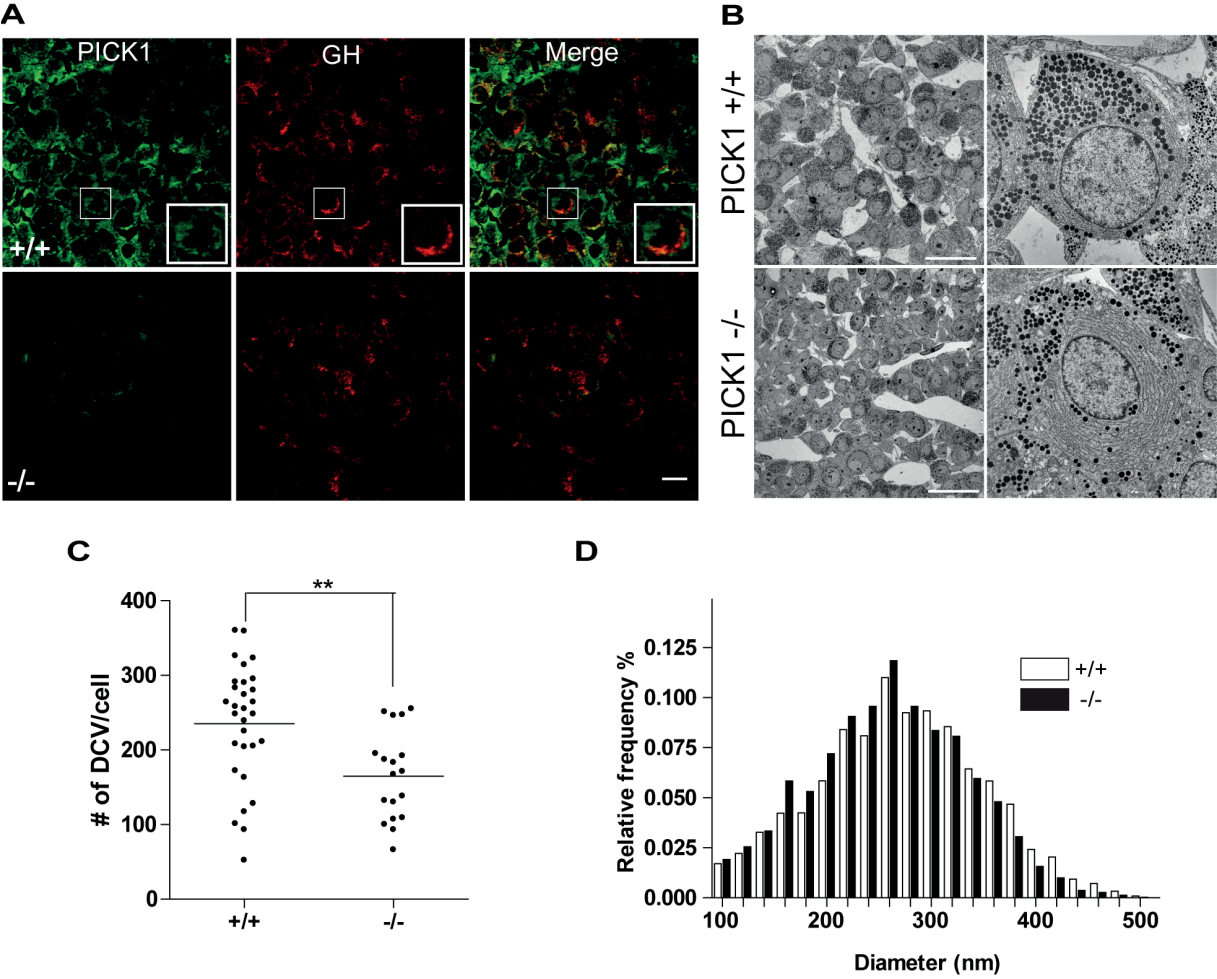
PICK1 deficiency leads to loss and altered localization of DCVs in the pituitary. (A) PICK1 is expressed in GH-producing cells of the pituitary. Confocal images of 15 µm slices from the pituitary of WT (+/+) (top) and PICK1-deficient mice (−/−) (bottom), immunostained for PICK1 (green) and GH (red). Insets show cells with characteristic intense arc-shaped distribution of GH and partial overlap with PICK1. Scale bar, 10 µm. Pictures are representative of stainings of seven pituitaries from WT and PICK1-deficient mice. (B) The number of DCVs is reduced in pituitary cells from PICK1-deficient mice. Transmission electron micrographs of uranyl acetate and lead citrate-stained ultrathin slices from the pituitary of WT (+/+) (top) and PICK1-deficient mice (−/−) (bottom). Scale bars, left, 20 µm. Pictures are representative of several pictures from five WT (+/+) and four PICK1-deficient (−/−) mice. (C) Quantification of the number of DCVs in 26 cells from WT (+/+) and 29 cells from PICK1-deficient (−/−) mice. **Average significantly different from control, *p* = 0.0022 in Student's *t* test. (D) Size distribution of DCVs in GH cells of the pituitary is unaltered in PICK1-deficient mice. The data show the relative frequency of different sizes of DCV in cells from PICK1-deficient mice (black) (*n* = 3,798) and in cells from WT mice (white) (*n* = 7,021).

To investigate the cellular basis for the metabolic phenotype of PICK1-deficient mice, we performed an immunohistochemical analysis of pituitaries from WT and PICK1-deficient mice. Intense PICK1 immunoreactivity was observed in a large fraction of WT pituitary cells (Figure 5A). Around half of the PICK1-positive cells were also positive for GH, while essentially all GH-positive cells showed concomitant PICK1 staining (Figure 5A). In individual WT cells, the GH staining formed a dense, often arc-shaped, pattern. Interestingly, in pituitaries from PICK1-deficient mice, the GH staining appeared less intense and less well organized, with no or only very few cells showing the WT pattern (Figure 5A). Electron microscopy confirmed the difference in intracellular structure of the GH-producing cells (identified by their characteristic architecture [30]). In the PICK1-deficient GH cells, we observed a reduction in the number of secretory dense core vesicles (DCVs) compared to WT cells (Figure 5B–C). The remaining vesicles were located at the cellular periphery (Figure 5B) with a possible slight decrease in the average size of the vesicles (Figure 5D). These observations further support that the GH pool is reduced in PICK1-deficient mice.

To assess whether PICK1 also was expressed in other endocrine cells of the pituitary, we performed immunostainings for prolactin and ACTH. A large proportion of cells with ACTH immunoreactivity were positive for PICK1 and, like GH in GH-producing cells, the ACTH immunosignal formed a circular pattern around the nuclei (Figure S3). For prolactin-positive cells, we also observed PICK1 immunosignal in a fraction of the cells, but we also observed cells that were positive for prolactin but showed no detectable PICK1 immunoreactivity (Figure S3). Thus, PICK1 expression is not restricted to GH-producing cells of the pituitary, coherent with a putative role of PICK1 in secretion of other pituitary hormones.

### PICK1 Is Expressed in GH-Producing GH1 Cells and Is Critical for GH Storage

**Figure 6:**
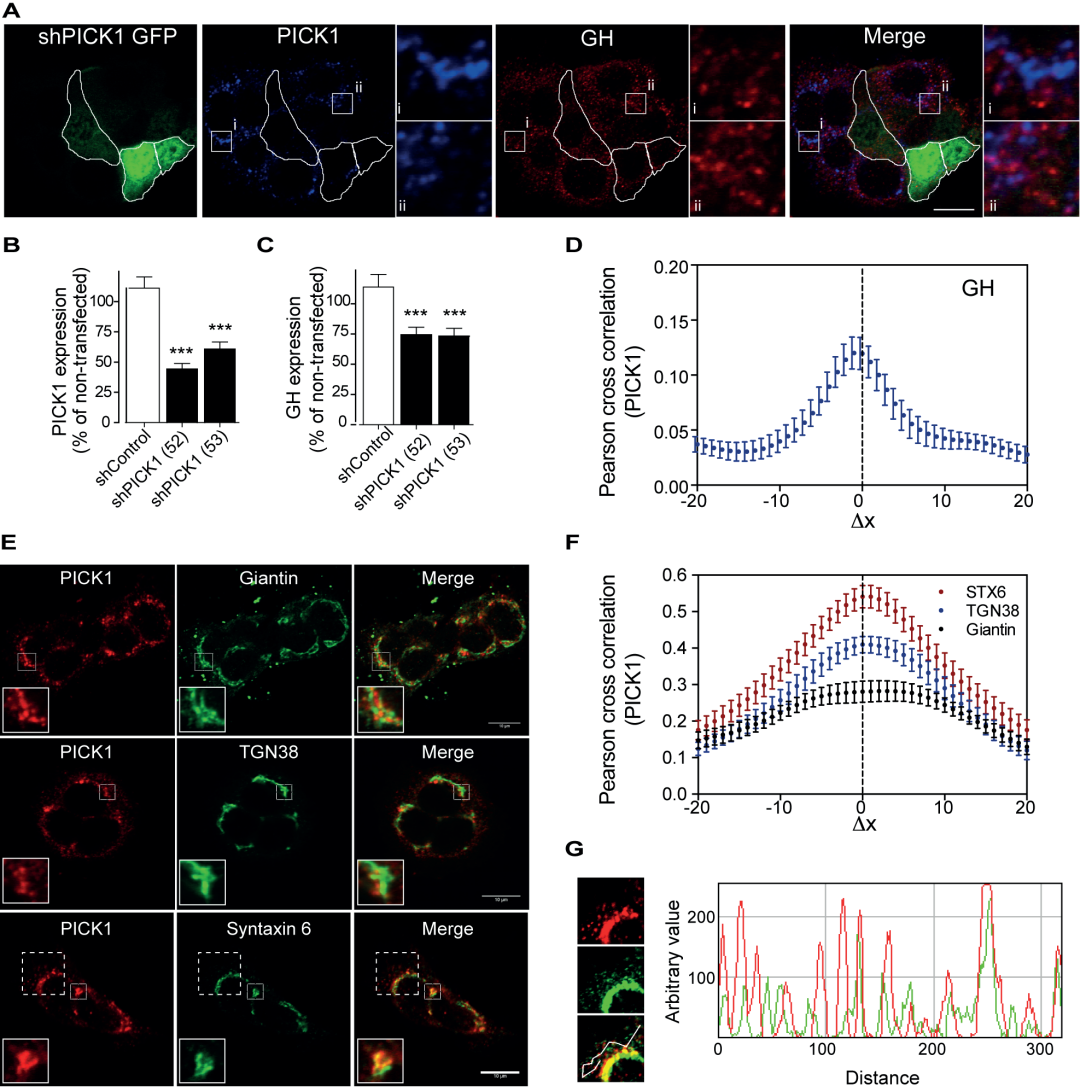
PICK1 is localized to vesicles exiting the Golgi in cultured GH1 cells, and knockdown of PICK1 reduces GH immunosignal in GH1 cells. (A) PICK1 is expressed in GH-producing GH1 cells, and PICK1 knockdown reduces GH content. Confocal images of GH1 cells immunostained for PICK1 (blue) and GH (red). Cells transfected with shRNA against PICK1 are identified by obligate coexpression of green fluorescent protein (EmGFP) (green) and are outlined. Insets (squares) show area with (i) no colocalization of PICK1 and GH and (ii) partial colocalization of PICK and GH. Scale bar, 10 µm. (B and C) Quantification of PICK1 immunosignal (B) and GH immunosignal (C) in GFP-positive cells transfected with either of two different shRNAs against PICK1 (52 and 53). Data are % of signal in surrounding non-transfected cells (mean ± SE) for control shRNA (shControl) (*n* = 48), shPICK1 (52) (*n* = 69), and shPICK1 (53) (*n* = 75). ***p<0.001, compared to shControl, Mann–Whitney rank sum test. (D) Quantification of the PICK1 co-localization with GH using Van Steensel's cross-correlation function, which reports the Pearson cross-correlation as a function of the relative movement of the two channels with respect to each other. The low but sharp peak close to Δx = 0 indicates partial but specific co-localization. (E) Co-localization of PICK1 with Giantin, TGN38, and syntaxin 6. Confocal laser scanning micrographs of GH1 cells immunostained for endogenous PICK1 (red) and the cis-Golgi marker giantin (top), the trans-Golgi (TGN) marker TGN38 (middle), and the TGN/immature vesicle marker syntaxin 6 (bottom) (all in green). Insets highlight area where the PICK1 signal was either enclosed by, or closely associated with, the Golgi markers (giantin, top, and TGN38, middle) or partially co-localizing (syntaxin 6, bottom). (F) Quantification of PICK1 colocalization with giantin, TGN38, and syntaxin 6 using Van Steensel's cross-correlation function. The broad peak for giantin is characteristic for large adjacent structures, whereas the high and sharp peak close to Δx = 0 for syntaxin 6 indicates specific co-localization. TGN38 is intermediate between these distributions, suggesting a partial overlap of the structure with PICK1. Co-localization was quantified for 10–20 cells from three independent experiments, and data are means ± SE. (G) Dashed box (left) from (E, Bottom) with magnification and gain adjusted for visualization of the punctate staining of PICK1 (red) and syntaxin 6 (green). Intensity profile (right) through several punctae (white line in lower, merged picture on the left) shows peaks corresponding to punctae for both PICK1 and syntaxin 6. All immunocytochemistry data were obtained from at least three independent experiments, and the cells are representative of multiple cells imaged in each experiment.

To exclude that the reduced GH pool was due to a defect in hypothalamic regulation, we studied GH1 cells, a GH-secreting cell line [31]. Immunostainings of these cells revealed a strong PICK1 signal (Figure 6A). To test if PICK1 is critical for the GH level in GH1 cells, we used shRNA to knock down PICK1 expression. Knockdown of PICK1 significantly decreased GH immunoreactivity, and thereby conceivably GH storage in shRNA transfected cells, which were identified by their obligate coexpression of EmGFP (Figure 6A–C). A control shRNA construct neither decreased PICK1 nor GH immunoreactivity (Figure 6B–C). Note that because only ∼5% of the GH1 cells were transfected with the shRNA constructs, it was not possible to assess PICK1 knockdown by, for example, western blotting or the effect on GH levels by ELISA. We should also note that PICK1 deficiency is unlikely to cause enhanced degradation of GH via the lysosomal pathway. Knockdown of PICK1 did not enhance co-localization of GH with lysosomal markers such as lysotracker and LAMP1, and overnight incubation of PICK1-depleted GH1 cells with an inhibitor of lysosomal degradation (leupeptin) did not rescue the GH immunosignal (unpublished data).

To test if PICK1 deficiency in other isolated endocrine cell lines depletes the secreted hormones, we analyzed the effect of PICK1 knockdown in the ACTH-producing AtT-20/D16v-F2 cell line (ATT20) [32]. In accordance with our findings for GH in GH1 cells, we observed a strong PICK1 immunosignal, and shRNA-mediated knockdown of PICK1 expression significantly reduced the ACTH immunosignal in these cells (Figure S4).

The reduced number of GH-containing DCVs in the pituitary of PICK1-deficient mice, together with reduced GH immunoreactivity in GH1 cells upon PICK1 knockdown, suggest that PICK1 might be directly involved in the biogenesis of GH-containing secretory vesicles. However, although we observed some colocalization of PICK1 with GH punctae in the GH1 cells (Figure 6A, inset ii, and Figure 6D), the PICK1 staining did only in part localize to the GH-positive vesicles and was also seen in juxtanuclear clusters (Figure 6A). The transferrin receptor, a marker of the early endocytic and recycling pathway, did not co-localize with these juxtanuclear PICK1 clusters, nor did the lysosomal marker LAMP1 (Figure S5A–B). To quantify the co-localization we applied Van Steensel's cross-correlation analysis, in which the Pearson cross-correlation is calculated as the signal from one channel (i.e., blue for PICK1) was shifted relative to the signal from the other channel (i.e., green for GH) in the x-direction pixel by pixel. In the Van Steensel's cross-correlation function, the Pearson cross-correlation is then plotted against Δx [33]. Consistent with partial but specific co-localization between PICK1 and GH, the cross-correlation function peaked at Δx = 0 with a value of ≈0.13 (Figure 6D). In contrast, we observed essentially no cross-correlation with transferrin or LAMP1 (Figure S5D).

We then tested whether PICK1 colocalized with markers for the entire Golgi (58K), the cis-Golgi (GM130, giantin), or the trans-Golgi network (TGN38). Although we did observe some overlap, the PICK1 staining generally displayed a more punctate pattern than the Golgi markers. Moreover, the PICK1 signal was preferentially enclosed by, or closely associated with, the Golgi markers rather than directly co-localizing with them (Figures 6E and S6A–B). Cross-correlation analysis confirmed these observations and revealed the highest maximum cross-correlation value (∼0.4) for TGN38 compared to ∼0.2 for giantin that also displayed a strongly broadened peak, consistent with adjacent localization of PICK1 and giantin with little true overlap (Figure 6F). A similar pattern of immunoreactivity was seen in *Drosophila* brains in which PICK1 immunoreactivity associated closely both with the trans-Golgi marker golgin-97 (Figure S6C) and with cis-Golgi markers [20] without truly overlapping. Moreover, we observed that PICK1 immunoreactivity surrounded zones of high dILP2 immunoreactivity in dILP-producing cells, with only partial overlap between the two signals (Figure S6D).

### PICK1 Is Localized to Immature Vesicles Exiting the TGN and PICK1 Tubulates Lipid Vesicles in Vitro

The strong PICK1 immunosignal adjacent to the Golgi led us to investigate the co-localization with markers of vesicles that either enter or exit the Golgi. We observed a noticeable co-localization with ERGIC53, a marker of the ER-Golgi intermediate compartment (ERGIC) (Figure S5C–D). A more striking co-localization was observed for syntaxin 6, a marker of immature secretory vesicles leaving the TGN (Figure 6E) [34]. When focusing on the punctate staining, it was possible to identify many punctae positive for both PICK1 and syntaxin 6 (Figure 6G). Furthermore, by staining simultaneously for PICK1, GH, and syntaxin 6, we were able to identify punctae and thus presumably early, immature vesicles positive for PICK1, syntaxin 6, and GH (Figure S6E–F). Cross-correlation analysis of the syntaxin 6/PICK1 staining substantiated the strong co-localization; that is, the cross-correlation function peaked essentially at ΔX = 0 with a maximum value of 0.55. Importantly, this suggests stronger co-localization between PICK1 and syntaxin 6 than between PICK1 and the Golgi markers (Figure 6F).

**Figure 7:**
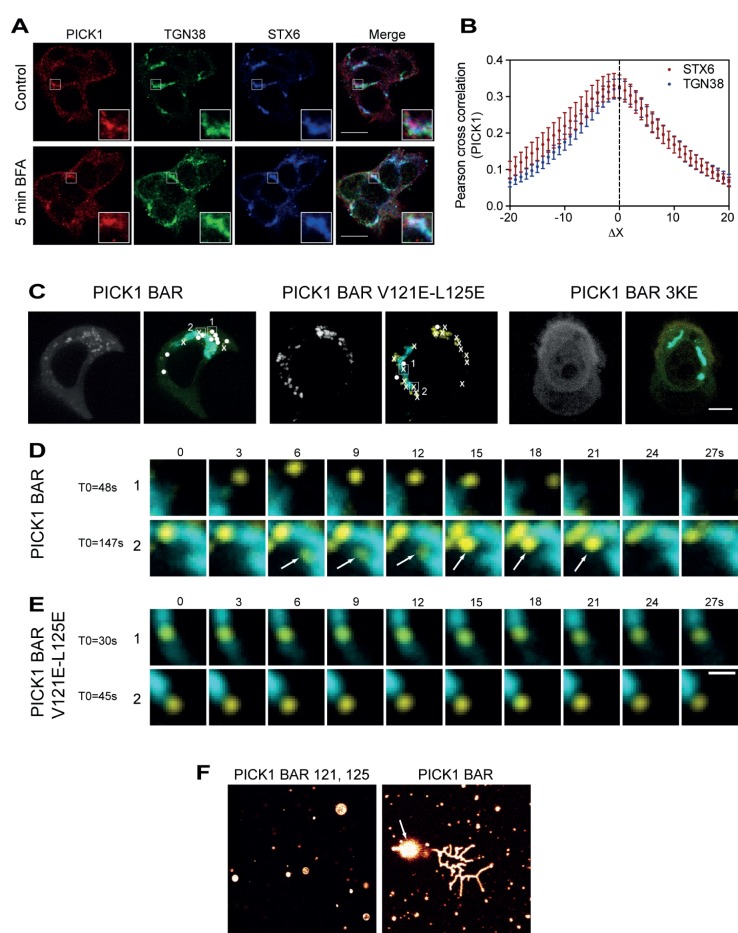
PICK1 associates transiently with the Golgi compartment and is capable of tubular deformation of liposomes in vitro. (A) Brief (5 min) brefeldin A treatment traps PICK1 in the trans-Golgi. Confocal images of GH1 cells immunostained for PICK1, and the trans-Golgi marker TGN38, and the trans-Golgi/immature vesicle marker syntaxin 6 before (top) and after 5 min BFA treatment (bottom). Panels show from left signal from immunolabeled PICK1 (Alexa Fluor 568 signal), signal from TGN38 (Alexa Fluor 488 signal), signal from syntaxin 6 (Alexa Fluor 647 signal), and overlay of the three channels. Insets highlight an area with overlapping localization of PICK1 and syntaxin 6 but with PICK1 adjacent to TGN38 (top) and colocalization of PICK1 with both syntaxin and TGN38 (bottom). (B) Quantification of the PICK1 colocalization with TGN38 and syntaxin 6 after 5 min BFA treatment using Van Steensel's cross-correlation function, which reports the Pearson cross-correlation as a function of the relative movement of the two channels with respect to each other. Both syntaxin 6 and TGN38 show a sharp peak of similar height close to Δx = 0, indicating specific co-localization. Co-localization was quantified for 10–20 cells from three independent experiments, and data are means ± SE. Note that the drop in peak values compared to no BFA (Figure 6F) likely reflects the increased diffuse localization of both markers after BFA treatment. (C) Fast time-lapse dual color live confocal imaging showing transient association of YFP-PICK1 BAR with the Golgi marker GalT-Cerulean. Images are maximal intensity projections of representative time-lapse series for YFP-PICK1 BAR (left), YFP PICK1 BAR V121E-L125E (middle), and YFP-PICK1 BAR 3KE (right). The gray scale projections (left images) show the YFP channel alone, indicating highly dynamic behavior of YFP PICK1 BAR (left) and more static behaviour of YFP-PICK1 BAR V121E-L125E (middle) punctae, whereas no punctae are seen for YFP-PICK1 BAR 3KE (right). The dual color projections (right images, YFP channel in yellow, Cerulean channel in blue) show that most of the activity for YFP-PICK BAR (left) is lining the Golgi, whereas YFP-PICK1 BAR V121E-L125E (middle) shows activity throughout the cell. Circles indicate transiently appearing punctate structures (lasting for less than 10 frames, ∼30 s). Crosses indicate stable punctate structures lasting for more than 10 frames. (D) Time-lapse series of two transient punctate structures of YFP-PICK1 BAR, and (E) two stable punctate structures of YFP-PICK1 BAR V121E-L125E. Scale bar, large images 5 µm, time lapse 1 µm. (F) Tubulation of surface-immobilized artificial giant membrane vesicles by the PICK1 BAR domain. Purified and Alexa Fluor 488–labeled GST-PICK1 BAR or GST-PICK1 BAR V121E-L125E (control) with impaired membrane binding capacity. Protein was incubated at room temperature with surface immobilized and fluorescently labeled vesicles before confocal fluorescent imaging. No effect was seen for the mutant, whereas massive tubulation was seen with GST-PICK1 BAR. Arrow indicates a budding vesicle from a single GV.

We next tested how PICK1 localization was affected by brefeldin A (BFA), an Arf1 inhibitor, which acutely arrests vesiculation at the Golgi [35]. Interestingly, a brief (5 min) treatment with BFA enhanced the apparent co-localization of PICK1 with TGN38 relative to syntaxin 6 (Figure 7A). Cross-correlation analysis of these data showed that in the absence of BFA the cross-correlation between the PICK1 and syntaxin 6 immunosignal was clearly higher than that between PICK1 and TGN38, whereas the cross-correlation signal was essentially identical after BFA treatment (Figure 7B).

Note that after BFA treatment the cross-correlation maximum was lower for both TGN38 and syntaxin 6 (compare to Figure 6F). This is most likely a consequence of an increased diffuse staining of both TGN38 and syntaxin 6 as expected upon BFA treatment (Figure 7A). Summarized, the data indicate that BFA traps PICK1 in the TGN and thus that PICK1 might be recruited to the TGN before localizing to Golgi proximal structures. This observation would be consistent with a role of PICK1 in budding of immature vesicles from the Golgi and thereby a function that parallels the role of other membrane-sculpting BAR domain proteins in budding processes such as clathrin-mediated endocytosis [12],[13].

To provide further evidence that PICK1 is recruited to the TGN to participate in the vesicle budding process and to investigate the kinetic behavior of PICK1, we first expressed in GH1 cells PICK1 tagged N-terminally with GFP (green fluorescent protein). Unfortunately, overexpressed GFP-PICK1 localized diffusely in GH cells consistent with the previously described autoinhibition of the PICK1 BAR domain [18]. However, truncation of the N-terminal PDZ domain relieves this autoinhibition, and indeed YFP-PICK1 BAR showed punctate localization in GH cells, with many punctae lining the Golgi compartment (identified by expression of the Golgi marker GalT, β1,4-galactosyltransferase) [36] tagged with cerulean fluorescent protein (GalT-Cerulean) (Figure S7). In contrast, a YFP-PICK1 BAR mutant with strongly reduced lipid binding capacity (3KE) [17],[18] showed diffuse localization in the cells (Figure S7). Live confocal imaging of cells co-expressing YFP-PICK1 and GalT-Cerulean revealed a highly dynamic behavior of the YFP-PICK1-positive punctae, in particular in the area lining the Golgi compartment, as illustrated by the maximal intensity projection of the time series (Figure 7C). In addition, we frequently observed transient events of punctate structures appearing transiently at the edge of the Golgi compartment. In Figure 7C, events lasting for less than 10 frames (∼30 s) are indicated by white dots, whereas stable punctate structures (>10 frames) are indicated by crosses (see also Movie S1). Of the transient events, seven out of 10 occurred at the edge of the Golgi compartment, and two of these events are shown as time series (1 and 2) in Figure 7D. The punctate structures appeared in many cases to originate from protrusions from the Golgi, suggesting that YFP-PICK1 BAR associates with vesicles budding from the Golgi apparatus (Figure 7D). Whether PICK1 dissociates from the newly formed vesicles within the 30 s time frame or whether the vesicles diffuse out of the plane could not be determined. Interestingly, introduction of two negative charges on the hydrophobic face of a putative N-terminal helix of the PICK1 BAR domain (PICK1 BAR V121E-L125E) did not change the punctate localization (Figures S7 and 7C); however, most punctae appeared stable (indicated by crosses in Figure 7C) and we did not see any transient events at the edge of the Golgi during the time lapses (Figure 7E and Movie S2). This suggests that V121E-L125E unlike the 3KE mutant is capable of localizing to and clustering at the Golgi but unable to dissociate from it. Notably, it was recently suggested that N-terminal amphipathic helices in BAR domains might facilitate fission events [37]. Summarized, the data demonstrate that the PICK1 BAR domain gets recruited to the Golgi and associates with vesicles that bud from the Golgi network. This further substantiates the idea that PICK1 is critical during the early stages of secretory vesicle formation.

We next asked whether the PICK1 BAR domain is capable of sculpting membranes and thus potentially contributes directly to the budding process. We incubated *in vitro* the isolated and purified PICK1 BAR domain with surface-immobilized artificial giant membrane vesicles. Confocal imaging showed that the PICK1 BAR domain indeed pulled tubular structures from the vesicles, whereas PICK1 BAR V121E-L125E had no observable effect (Figure 7F). Unfortunately, we could not test the effect of PICK1 BAR 3KE because it expressed poorly in the bacteria, making it impossible to obtain sufficient amount of pure protein for the experiment (unpublished data).

### PICK1 Exists in a Complex with ICA69 and PICK1-Deficiency Abolishes ICA69 Protein Expression in Mice and *Drosophila*

**Figure 8:**
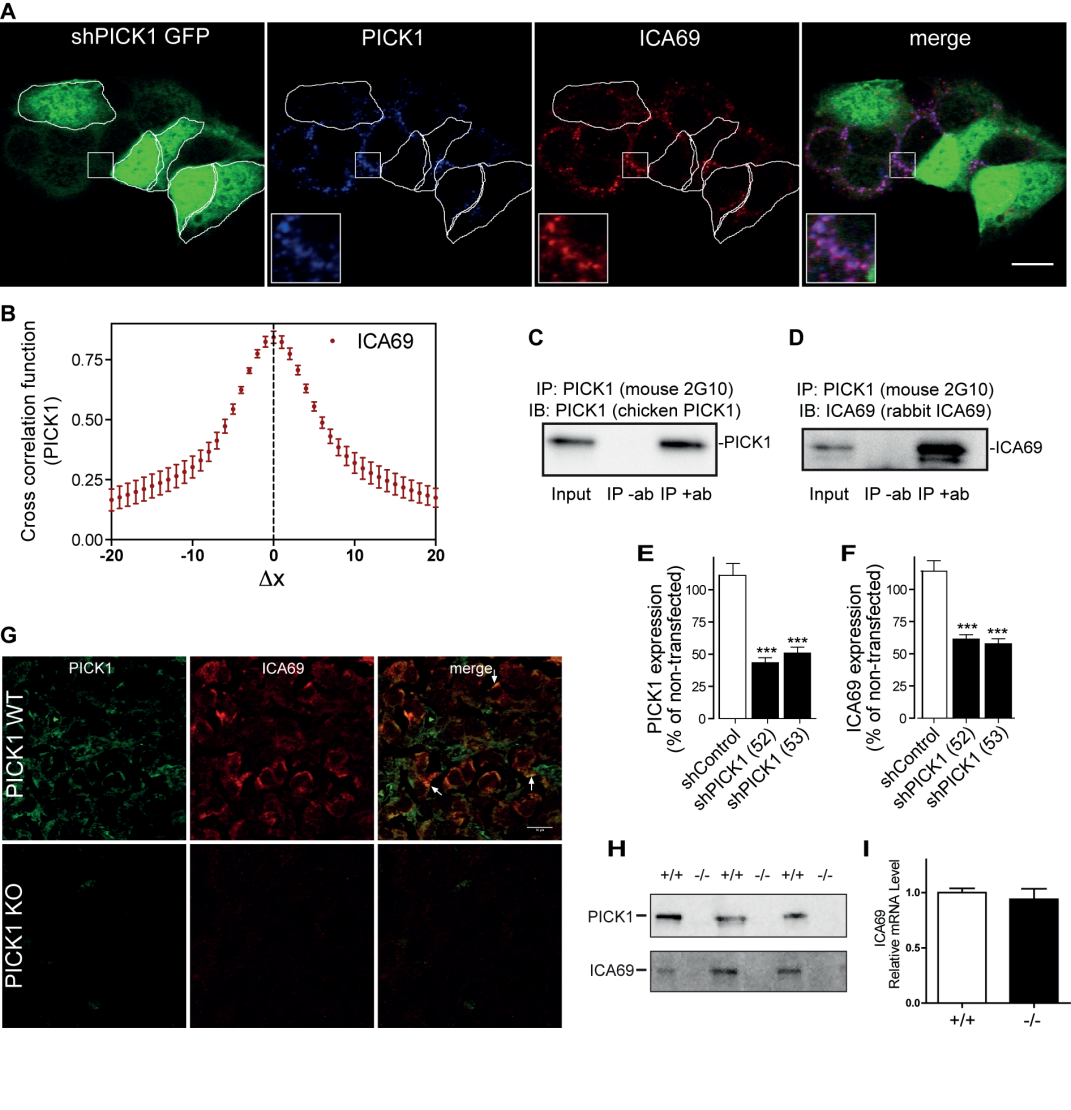
ICA69 expression is dependent on PICK1, and the two proteins co-localize and co-immunoprecipitate. (A) PICK1 co-localizes with ICA69. Confocal images of GH1 cells immunostained for PICK1 and ICA69. Cells transfected with shRNA against PICK1 are identified by obligate co-expression of EmGFP and are outlined. Insets demonstrate extensive PICK1 and ICA69 colocalization. Pictures are representative of multiple cells imaged in three independent experiments. Scale bar, 10 µm. (B) Quantification of co-localization between ICA69 and PICK1 using Van Steensel's cross-correlation function, which reports the Pearson cross-correlation as a function of the relative movement of the two channels with respect to each other. In agreement with striking co-localization, the analysis revealed a sharp peak at ΔX = 0 with a maximum >0.8. Co-localization was quantified for 10–20 cells from three independent experiments, and data are means ± SE. (C) Western blotting with chicken anti-PICK1 antibody of immunoprecipitates obtained with mouse anti-PICK1 2G10 antibody in GH1 cells lysates. Left lane, lysate input; middle lane, no 2G10 antibody. (D) Co-immunoprecipitation of ICA69 with PICK1. Western blotting with rabbit anti-ICA69 antibody of immunoprecipitates obtained with mouse anti-PICK1 2G10 antibody in GH1 cells lysates. Left lane, lysate input; middle lane, no 2G10 antibody. (E and F) Quantification of immunosignal of ICA69 (B) and of PICK1 (C) in GFP-positive cells transfected with either of two different shRNAs against PICK1 (52 and 53). Data are percent of expression in surrounding nontransfected cells (means ± SE) for control shRNA (shControl) (*n* = 61), shPICK1(52) (*n* = 85), and shPICK1 (53) (*n* = 75). ***p<0.001 as compared to shRNA control, Mann–Whitney rank sum test. (G) ICA69 is expressed in GH-producing cells of the pituitary and co-localizes with PICK1 in WT mice but is absent in cells from PICK1-deficient mice. Arrows show examples of extensive PICK1/ICA69 co-localization in WT mice. Scale bar, 10 µm. Pictures are representative of stainings of seven pituitaries from WT and PICK1-deficient mice. (H) ICA69 immunoreactivity is essentially absent in PICK1-deficient mice. Western blots of pituitary extracts from WT and PICK1-deficient mice (three representative of each) using rabbit anti-ICA69 antibody and anti-PICK1 2G10 antibody. (I) Similar ICA69 mRNA levels in WT and PICK1-deficient mice. Quantification by RT-PCR of ICA69 mRNA in the pituitary from WT and PICK1-deficient mice. Mice of 35–38-wk-old were used. White bars, PICK1+/+: black bars, PICK1 −/−. Data are means ± SE (*n* = 7–10).

The cellular localization of PICK1 partially resembled that of the closely related BAR domain protein ICA69 [22], previously suggested to associate with PICK1 in neurons via BAR domain heterodimerization [19]. Indeed, ICA69 was expressed in GH1 cells and showed striking colocalization with PICK1 (Figure 8A) as also reflected in the cross-correlation analysis revealing a sharp peak at Δx = 0 with a maximum >0.8 (Figure 8B). Moreover, ICA69 and PICK1 co-immunoprecipitated in extracts from GH1 cells, supporting that the two proteins form a heterodimeric complex (Figure 8C–D). Interestingly, knockdown of PICK1 expression in GH1 cells caused a parallel robust decrease in the ICA69 protein level, as evidenced by a marked decrease in immunoreactivity (Figure 8A,E,F). We also assessed ICA69 expression in ATT20 cells, and similar to our observations in GH1 cells, there was significant ICA69 immunostaining overlapping with the PICK1 signal (Figure S8A). Furthermore, knockdown of PICK1 in ATT20 cells significantly reduced ICA69 immunoreactivity (Figure S8B–C).

**Figure 9:**
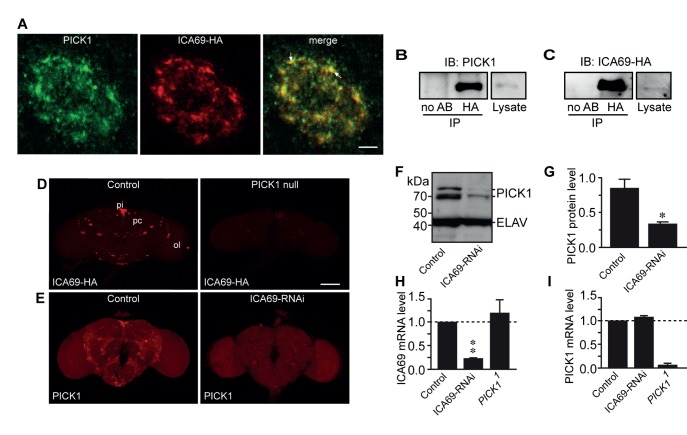
Interdependent expression of PICK1 and ICA69 in *Drosophila*. (A) Immunostainings for ICA69 and PICK1 in a single peptidergic neuron in the protocerebrum of adult *Drosophila* brain. HA-tagged ICA69 targeted to peptidergic cells (*c929-GAL4/+; UAS-ICA69-HA/+*) was detected in parallel with endogenous PICK1. Arrows indicate examples of PICK1/ICA69 co-localization. Scale bar, 10 µm. (B and C) *Drosophila* ICA69-HA co-immunoprecipitates PICK1 from fly head extracts. Flies were co-expressing ICA69-HA and PICK1 under the pan-neuronal elav-GAL4 driver (*elav-GAL4/+; UAS-ICA69-HA/UAS-PICK1A*). (B) Immunoprecipitation (IP) with anti-HA antibody and immunoblotting (IB) with anti-PICK1 antibody. Left lane, control without antibody; right lane, lysate. (C) Immunoprecipitation (IP) with rat anti-HA antibody (3F10) and immunoblotting (IB) with mouse anti-HA (16B12) antibody. Left lane, control without antibody; right lane, lysate. (D) Dependence of ICA69 expression on PICK1 expression in *Drosophila*. ICA69-HA immunoreactivity is shown in control brain (*c929-GAL4*, *PICK^1^/+; UAS-ICA69-HA/+*) and in PICK1-null mutant brain (*c929-GAL4, PICK^1^/PICK^2^; UAS-ICA69-HA/+*). pi, pars intercerebralis; pc, protocerebrum; ol, optic lobe. Pictures are representative of six fly brains stained in three experiments. (E) Dependence of PICK1 expression on ICA69 expression in *Drosophila*. PICK1 immunosignal in control brain without RNAi transgene (*ELAV-GAL4/UAS-DCR2;;TM3/+*) and in brain with pan-neuronal expression of ICA69 hairpin RNA (*ELAV-GAL4/UAS-DCR2;;UAS-ICA-RNAi/+*) (ICA69-RNAi). Scale bar, 100 µm. Pictures are representative of six fly brains stained in three experiments. (F) Western blotting of head extracts from control and ICA69 RNAi flies. (G) Quantification of the data in (F). Protein levels are normalized to ELAV. Data are means ± SE, *n* = 6, *p*<0.05. (H and I) Quantification by RT-PCR of ICA69 mRNA (H) and PICK1 mRNA (I) levels in heads from *ICA69* knockdown (ICA69-RNAi), *PICK1^1^*, and control flies. Data are means ± SE, *n* = 6 for control and ICA69-RNAi, *n* = 4 for *PICK1^1^*, *p<0.05, **p<0.01.

These observations in GH1 cells led us to investigate ICA69 expression and localization in the pituitary of WT and PICK1-deficient mice by immunohistochemistry. In WT mice, the ICA69 immunosignal clearly overlapped with the PICK1 signal both at the cellular and subcellular levels (Figure 8G). Strikingly and consistent with our findings in GH1 cells, we were unable to detect ICA69 expression in PICK1-deficient mice by immunohistochemistry (Figure 8G). This lack of ICA69 protein expression was confirmed by Western blotting of pituitary extracts from PICK1-deficient mice (Figure 8H) and was not caused by reduced transcription (Figure 8I). To assess whether PICK1-dependency of ICA69 expression is evolutionary conserved, we expressed *Drosophila* ICA69 tagged with hemagglutinin (HA) in peptidergic neurons of WT and PICK1 null flies. In WT neurosecretory neurons, the immunoreactivities of exogenous ICA69-HA and endogenous PICK1 overlapped extensively (Figure 9A). In flies co-expressing ICA69-HA and PICK1A under the control of the pan-neuronal elav-GAL4 driver, we were also able to co-immunoprecipitate the two proteins (Figure 9B–C). Furthermore, the ICA69 immunosignal was dramatically reduced in PICK1-null flies (Figure 9D). We next tested the converse possibility that the PICK1 protein level depends on the ICA69 level, by knocking down ICA69 expression with a UAS-ICA69-RNAi construct. ICA69-RNAi markedly reduced endogenous PICK1 staining, with no change in control brains (Figure 9E). Likewise, Western blots showed a severe drop in the PICK1 immunosignal in ICA69-RNAi flies compared to controls (Figure 9F–G). RT-PCR analysis verified reduced ICA69 mRNA levels in ICA69-RNAi flies, whereas PICK1 mRNA levels were unaffected (Figure 9H). Conversely, in PICK1-null flies ICA69 mRNA was not reduced, whereas PICK1 mRNA was hardly detectable (Figure 9I). We finally tested whether the mutual interdependency of PICK1 and ICA69 expression could be reproduced in GH1 and ATT20 cells. This was indeed the case, as shRNA-mediated knockdown of ICA69 led to a parallel decrease in apparent PICK1 expression in both cell lines (Figures S8D–F and S9A–C).

Together, the data suggest that PICK1 might exert its function in a heterodimeric complex with ICA69 and that the two proteins stabilize one another. The ability of PICK1 to stabilize expression of ICA69 was supported by expression of the proteins in a non-endocrine cell line. Flp-In T-REx 293 cells stably expressing HA-tagged ICA69 (HA-ICA69) in a tetracycline-dependent manner (Flp-In T-REx 293 HA-ICA69 cells) were transfected with YFP-PICK1 or empty plasmid before induction of the cells with tetracycline for 24 h. Western blotting revealed several folds higher ICA immunoreactivity in YFP-PICK1-expressing cells compared to control cells (Figure S10).

**Figure 10:**
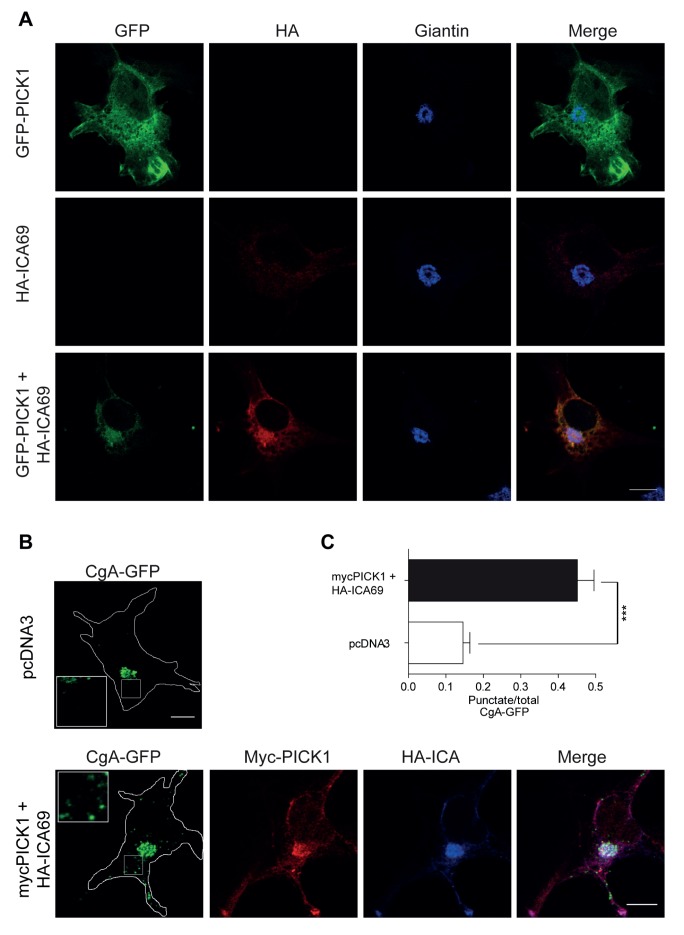
PICK1 and ICA69 co-localize in the Golgi compartment of COS7 cells and promote punctate distribution of GFP-tagged chromogranin A (CgA-GFP) in the cytosol. (A) Confocal images of COS-7 cells transfected with GFP-PICK1 (top), HA-ICA69 (middle), or GFP-PICK1 and HA-ICA69 (bottom). From left, the channels show GFP-PICK1 in green, HA-ICA69 in red, and endogenous giantin in blue. Upon co-expression, GFP-PICK1 and ICA69 co-localize with giantin in the Golgi compartment. Scale bar, 10 µm. (B) Confocal images of COS-7 cells transfected with CgA-GFP and pcDNA3 (top) or together with mycPICK1 and HA-ICA69 (bottom). From left, the green channel shows CgA-GFP, the red channel shows mycPICK1, and the blue channel shows HA-ICA69. Insets illustrate the marked increase in punctuate CgA structures in cells co-transfected with mycPICK1 and HA-ICA69. Outlines of the cells are shown in the green channel in white. Scale bar, 10 µm. Images are representative of *n* = 50 (CgA-GFP) and *n* = 44 (CgA-GFP+mycPICK1+HA-ICA69) cells from three independent sessions. (C) Quantification of the punctuate CgA-GFP signal relative to the total CgA-GFP signal as a measure of the efficiency of Golgi exit. ***p<0.001 in Student's *t* test.

Finally, we investigated whether PICK1 together with ICA69 might have a general role in the formation of secretory vesicles. Previously, it was shown that expression of chromogranin A (CgA) in non-endocrine cells can lead to formation of dense core-like granules in non-endocrine cells, consistent with the functional importance of chromogranins in secretory vesicle formation [1],[6],[7]. If, as suggested by our data, PICK1 together with ICA69 function to facilitate the formation of secretory vesicles, we hypothesized that co-expression of PICK1 and ICA69 together with CgA should promote formation of CgA-positive vesicles in non-endocrine cells. First, we expressed PICK1 fused to GFP (GFP-PICK1) and HA-ICA69 either alone or together in COS7 cells. When expressed alone, the resulting GFP-PICK1 and HA-ICA69 immunosignals were relatively uniformly distributed in the cytoplasm showing little co-localization with the Golgi marker giantin (Figure 10A). However, when expressed together, the GFP-PICK1 and HA-ICA69 immunosignals clustered around the nucleus and showed marked overlap with the giantin immunosignal (Figure 10A). When expressed alone in COS7 cells, CgA fused to GFP (CgA-GFP) localized primarily to a Golgi-like juxtanuclear compartment with little localization to punctate structures in the cytoplasm (Figure 10B), a localization that was confirmed in giantin co-stainings (unpublished data). However, when GFP-PICK1 and HA-ICA69 were expressed together with CgA-GFP, we observed a remarkable redistribution of CgA-GFP. Now, CgA-GFP localized to a much higher degree to punctate structures in the cytoplasm, possibly representing CgA containing vesicles (Figure 10B). Quantification showed that the punctate signal relative to total signal increased almost 3-fold (Figure 10C), consistent with the concept of a direct role of the PICK1/ICA69 complex in formation of CgA containing vesicles. Notably, the majority of the immunosignal from GFP-PICK1 and ICA69 was seen in the Golgi-like juxtanuclear compartment and little co-localization of GFP-PICK1 and HA-ICA69 with CgA in vesicular structures. Importantly, this is agreement with our observation for GH-positive vesicles in GH-1 cells; that is, PICK1 and ICA69 localize primarily to the early secretory vesicles and not necessarily to the mature vesicles.

### Altered Expression of PICK1 in Models of Metabolic Diseases

**Figure 11:**
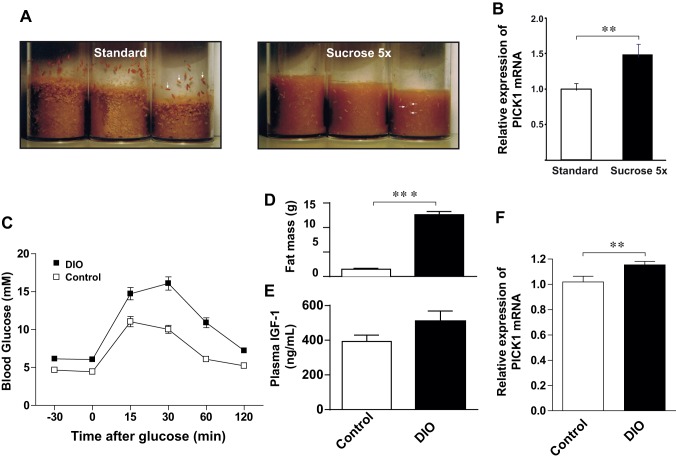
Up-regulation of PICK1 expression in *Drosophila* under HSD that induces peripheral dILP resistance. (A) Delayed larval development in response to HSD that induces peripheral dILP resistance [38],[39]. WT flies were allowed to deposit eggs on an apple juice plate for 6 h. On the following day, first instar larvae hatched from the eggs were transferred to vials either containing food with the standard amount of sucrose (left) or five times that amount (right). Photographs were taken 6 d following oviposition. Note that, when all the larvae in the vials containing standard food have already left the food, completed the wandering stage, and commenced pupariation (left, vertical arrows), the larvae in the vials with 5× sucrose have yet to leave the food to commence the wandering stage (right, horizontal arrows). (B) Quantitative RT-PCR was performed on pooled heads from fully developed adult female flies reared on standard food (*n* = 9 samples) or food with five times the normal amount of sucrose (*n* = 7). Data show relative change (means ± SE). **p<0.01. (C) Impaired glucose tolerance in high-fat-diet-induced obese C57BL mice (DIO). OGTT was performed on DIO mice (black squares) and littermate controls fed on a normal chow diet (white squares). After 16 h fasting, the DIO mice displayed increased glucose level compared to control mice (6.1±0.3 mM versus 4.4±0.2 mM; *p* = 0.0006), and after the glucose load, the blood glucose level was significantly higher at any time point (*p*≤0.001). (D) The DIO mice also displayed significantly higher fat mass compared to control mice (12.6±0.6 g versus 1.48±0.21 g; ***p≤0.0001). (E) Total plasma level of IGF-1 in DIO mice (510±59 ng/mL) compared to control mice (393±37 ng/mL) (*p* = 0.11). (F) The relative expression of PICK1 mRNA assessed by RT-PCR was increased in the pituitaries of DIO mice. All measurements were performed on *n* = 14 high-fat-diet-treated mice and 14 mice fed a normal chow diet and data are expressed as means ±SE.

Given the profound metabolic changes in PICK1-deficient flies and mice, we wanted to assess whether PICK1 might be linked to metabolic pathophysiology. First, we used a *Drosophila* model of T2D, in which larvae are grown on a high-sugar diet (HSD). As a consequence of the HSD, they develop hyperglycemia, insulin resistance, and other hallmarks of human T2D. Moreover, larval development under HSD is considerably delayed due to the resistance of the peripheral tissues to the dILPs, which control *Drosophila* growth and sugar homeostasis (Figure 11A) [38]–[40]. In both flies and mammals, peripheral insulin resistance is associated with a compensatory increase in production and secretion of insulin peptides [38]–[40]. If PICK1 is important in the biogenesis of insulin/dILP-containing vesicles, PICK1 expression might be altered in response to HSD. Confirming earlier reports [38]–[40], we found that rearing larvae on HSD markedly delayed their development. More importantly, when assessing PICK1 mRNA expression in the heads of the adult flies, we observed ∼50% up-regulation of PICK1 mRNA in flies reared on HSD during their development as compared to control flies (Figure 11B).

To study the involvement of PICK1 in a mammalian model of metabolic syndrome, we used adult (18-wk-old) male mice fed a 60% high-fat diet for 12 wk. This model (diet-induced obesity, DIO is a well-described tool for studying human metabolic syndrome [41]. The deregulated glucose metabolism was verified by decreased oral glucose tolerance, increased fasting glucose, and increased fat mass (Figure 11C–D) as well as increased basal and glucose-stimulated plasma insulin levels (basal insulin, 115±17 pg/mL in control mice and 1,110±170 pg/mL in DIO mice p<0.0001; glucose stimulated insulin, 1,830±190 pg/mL in control mice and 7,460±970 pg/mL in DIO mice, *p*≤0.0001). Because GH is secreted in a pulsatile manner, we measured plasma IGF-1 as marker of total GH secretion [25] and found a trend towards an increase in the DIO mice (Figure 11E). Interestingly, we also found that the PICK1 mRNA expression level in the pituitary was significantly up-regulated by ∼15% in the DIO mice (Figure 11F). Thus, PICK1 expression is both in flies and mice sensitive to changes in metabolic states that mimic the human metabolic syndrome.

## Discussion

The present study suggests that the membrane-sculpting BAR domain protein PICK1 serves an important and hitherto unknown role in endocrine physiology by controlling the efficiency of dense core secretory vesicle biogenesis in peptidergic endocrine cells. Our initial key observation is that flies and mice that are deficient in PICK1 display marked somatic growth retardation. In the flies, we can link this phenotype to absence of PICK1 from peptidergic neurons secreting somatotropic peptides functionally equivalent to GH and insulin in mammals. In the mice, we can link the phenotype to impaired storage and secretion of GH from the pituitary and likely also of insulin from the pancreas. At the cellular level, EM analysis of mouse pituitary showed that PICK1 deficiency leads to a prominent decrease in the pool of secretory vesicles. There was no apparent change in vesicle size and appearance, which is consistent with a simple decrease in the rate of secretory vesicle formation. To exclude possible effects of deficient hypothalamic regulation, we also knocked down PICK1 expression in GH1 cells, an immortalized GH-producing cell line. This procedure markedly reduced apparent GH levels, in agreement with our observations in the pituitary.

Co-localization studies suggested that PICK1 primarily is localized adjacent to the Golgi, and we observed strong co-localization with syntaxin 6 that marks immature vesicles budding from the TGN [34]. Our co-localization experiments were quantitatively strongly supported by application of Van Steensel's cross-correlation analysis [33], although we should note that despite the outcome of this analysis, the experiments do not exclude that PICK1 and ICA69 might reside in yet unidentified compartments as well. Nonetheless, it was striking to observe how a brief brefeldin A treatment enhanced co-localization with TGN, suggesting that PICK1 transiently associates with this compartment. In addition, we could show by live imaging of GH1 cells that the WT PICK1 BAR domain gets recruited to the Golgi and dynamically associates with vesicles that bud from the Golgi network. Together with the ability of the PICK1 BAR domain to tubulate membranes in vitro, these observations support a direct involvement of PICK1 in budding of GH containing vesicles from the TGN.

Our phenotypic analysis of PICK1-deficient mice demonstrated classical characteristics of GH deficiency, including decreased body weight and length, increased body fat accumulation, less lean body mass, reduced ghrelin-induced GH secretion, reduced liver weight, and decreased IGF-1 expression substantiated both by decreased plasma levels and lower mRNA expression in the liver [42]. An even more severe metabolic phenotype of PICK1-deficient mice was suggested by decreased glucose tolerance combined with impaired glucose-induced insulin secretion. These observations agree with those of Xia and colleagues showing impaired insulin secretion from β-cells in the pancreas of PICK1-deficient mice [29]. Xia and colleagues did not, however, observe increased insulin sensitivity. Furthermore, they found a small but significant increase in daily food and water intake of the PICK1-deficient mice [29], where we observed a tendency toward an increase. We have no immediate explanation for these apparent discrepancies other than they might be caused by slight differences in the experimental procedures and the fact that the PICK-deficient mice, although derived from the same knock-out strain, have been maintained separately. It is important to note that increased insulin sensitivity indeed is known to be associated with impaired GH function [27]. In further agreement with impaired GH function and increased insulin sensitivity, we identified an up-regulation of insulin receptor protein levels in the liver [43],[44].

To assess the importance of the GH deficiency for the metabolic phenotype, we administered GH to PICK1-deficient mice, and we observed an increase in lean body mass approaching WT levels within 2 wk. After the treatment, insulin sensitivity was similar to that of WT mice, and IGF1-mRNA expression levels were not significantly different from WT animals. In the glucose tolerance test, we saw no signs of recovery, suggesting that the decreased glucose-induced insulin secretion observed in the PICK-deficient mice is independent of the GH-deficient phenotype. On the other hand, GH treatment is known to be diabetogenic and could negatively affect the glucose tolerance [27]. It remains nevertheless an open question whether PICK1 is critical for secretory vesicle biogenesis in peptide-producing endocrine cells in general. In the pituitary, we observed a significant decrease also in prolactin content, but only a trend was seen for ACTH and TSH. However, knockdown of PICK1 in ACTH-producing ATT20 cells led to a significant decrease in apparent ACTH levels, showing that PICK1 might be important for ACTH secretion at least in isolated cells. A more general role of PICK1 in peptide hormone secretion is therefore possible, and it is important to keep in mind that in the PICK1 knock-out mice, adaptive changes occurring during development might compensate for the absence of PICK1 in some systems and not in others, maybe depending on the turnover rate of the secretory vesicle pool.

It is interesting that PICK1 deficiency does not completely deplete the pool of secretory vesicles in the GH-producing cells. This indicates that PICK1 serves primarily to accelerate vesicle formation, trafficking, and maturation. Such a role would also be compatible with previous studies stressing that other cellular components are important for driving TGN vesicle budding and formation. Cholesterol has been proposed to play a role via its ability to promote formation of membrane rafts on the cholesterol-enriched maturing face of the TGN [1],[5]. Moreover, chromogranins, which are present in secretory vesicles of all neuroendocrine cells, were suggested to constitute a driving force for vesicle budding [1],[6],[7]. Chromogranins aggregate together with prohormones at conditions corresponding to those inside the TGN (i.e., at low pH in the presence of Ca^2+^), and the budding process might be stimulated by associations of these aggregates with membrane rafts [1],[6],[7]. Remarkably, we could show that co-transfection of PICK1 and ICA69 promotes punctate localization and possibly vesicular storage of chromogranin in non-endocrine COS7 cells, supporting the interesting possibility that chromogranins operate together with the PICK1/ICA69 complex in the biogenesis of secretory vesicles. Hence, PICK1 might, together with ICA69, associate with cholesterol-enriched microdomains on the maturing face of the TGN and thereby impose the necessary bending of the lipid bilayer.

In the accompanying paper, Xia and colleagues propose that PICK1 and ICA69 are associated primarily with immature vesicles positive for proinsulin, while PICK1 alone is associated with mature insulin-positive vesicles [29]. We did not find evidence for this distinction in GH1 cells where the co-localization between the two proteins is remarkable, with a Pearson cross-correlation value >0.8. Possibly, there is a functional difference between the two different types of endocrine cells. An important difference is that biosynthesis of insulin involves conversion of proinsulin to insulin, which is not the case for GH. Of note, because the vesicle maturation process involves conversion of proinsulin to insulin, it is possible in insulin-producing cells to use the proinsulin level as a measure of secretory vesicle maturation, which cannot be done in GH-producing cells.

BAR domain proteins have previously been implicated in other budding processes such as clathrin-mediated endocytosis. Current insights suggest that at least four different BAR domain proteins including the FCHO1/2, SNX9, amphiphysin, and endophilins are involved. Recently, it was demonstrated that FCHO1/2 is required for induction of the initial membrane curvature and thereby for nucleation of clathrin-coated pit formation [12],[13]. In subsequent steps, amphiphysin is believed to play a role in membrane bending and dynamin recruitment to clathrin-coated pits, SNX9 in AP2 and dynamin binding, and endophilins in dynamin and synaptojanin recruitment [12],[13]. However, although both endocytosis and secretory vesicle biogenesis involve vesicle budding, there are also inherent differences between the two processes. For example, the putative driving force provided by a high density of prohormones and chromogranins inside the TGN is not available for endocytosis [1]. Thus, the present data extend the role of two BAR domain proteins to a vesicle-budding process that is different in nature from that of clathrin-mediated endocytosis. An interesting question is whether other BAR domain proteins are involved in addition to PICK1 and ICA69.

With the exception of a function in acrosome biogenesis [45], PICK1 has been known almost exclusively for its roles in regulating trafficking and phosphorylation of membrane proteins in the CNS [14]–[17],[46]. Hence, our data reveal a remarkable functional diversity of the PICK1 protein by identifying a so far unknown evolutionary conserved function. PICK1 is known particularly for regulating surface levels and subunit composition of AMPA-type glutamate receptors, and thereby for synaptic plasticity including both long-term depression (LTD) and long-term potentiation (LTP) as well as other types of plasticity [14]–[17],[46]–[50]. Despite the convincing evidence for these conclusions, it is interesting to consider that the role of PICK1 in synaptic plasticity might also depend on its role in controlling secretory vesicle biogenesis. PICK1 may be involved in storage and secretion of, for example, neurotrophic factors, such as BDNF (brain-derived neurotrophic factor), which are known to play an important role in regulating synaptic plasticity [51].

PICK1 co-localized and co-immunoprecipitated with the homologous BAR domain protein ICA69 that differs from PICK1 by not having an N-terminal PDZ domain [19]. We also observed that ICA69 protein expression was strongly dependent on PICK1 in mice, GH1 cells, ATT20 cells, and the *Drosophila* brain. In FlpIn 293 cells without a regulated secretory pathway, PICK1 enhanced ICA69 protein expression, adding further support to a direct stabilizing role of PICK1. In GH1 cells, ATT20 cells, and flies, we could demonstrate the reciprocal dependency; that is, knockdown of ICA69 reduced PICK1 protein expression. Altogether, the data support that PICK1 exerts its function in a heterodimeric complex with ICA69. This agrees with previous observations indicating heterodimerization of PICK1 with ICA69 in neurons [19]. Of interest, ICA69 was proposed to be involved in regulating secretion from neurosecretory cells [22] and was also shown to be an effector of the small GTPase Rab2 [52],[53]. Future experimental efforts should further clarify the functional impact of the PICK1/ICA69 interaction.

Because of the severe endocrine dysfunction and changed metabolic homeostasis that we observe in PICK1-deficient mice, we tested whether PICK1 expression might be altered in two metabolic disease models, one in flies [38]–[40] and one in mice [41]. In adult flies, reared during development on a HSD, PICK1 mRNA levels increase significantly, and correspondingly PICK1 mRNA levels in the pituitary increase in mice fed on a high-fat diet (DIO mice). We also measured plasma IGF-1 levels in the DIO mice as a marker of GH secretion and found a trend toward an increase. Interestingly, a significant increase in circulating IGF-1 levels was found in a recent study on obese mice introduced to a high-fat diet at a slightly younger age than the mice used on our study [54]. Furthermore, it should be noted that evaluation of the plasma level of free or total IGF-1 in obesity might be complicated by factors such as IGF binding proteins and fat mass [55]. Summarized, PICK expression appears sensitive to metabolic changes that characterize metabolic syndrome and/or T2D; hence, it is possible that PICK1 plays a role in the pathophysiological process of metabolic diseases or in a protective compensatory mechanism.

It is also striking that the phenotype of the PICK1-deficient mice resembles the clinical symptoms associated with human GH deficiency [42]. Congenital GH deficiency has an incidence of one in 4,000–10,000 children, of which the majority is expected to be caused by genetic factors. Nonetheless, the causative genetic defect has only been identified in a small fraction of these patients [42]. Variants of the PICK1 and perhaps ICA69 could very well be responsible for a subset of idiopathic GH-deficiency patients characterized by decreased somatic growth and increased fat accumulation. Finally, the identification of a BAR domain protein as a key player in controlling storage and secretion of GH and possibly other hormones should prove important for our general understanding of secretory processes and potentially lead to the development of new treatments for diseases characterized by impaired secretion of hormones.

## Material and Methods

### *Drosophila* Genetics and Molecular Biology

The *PICK1^1^* and *PICK^2^* null alleles are identical to *PICK1^ΔEP-147^* and *PICK1^ΔEP-197^*, respectively, which were generated by imprecise excision of the P-element EP2315, as described [20]. The precise excision allele *PICK1^ΔEP-213^*, generated in the same jump-out mutagenesis screen [20], was used as control (Figure 1). *Drosophila PICK1* is expressed as an A-form and a shorter B-form; the *P{w^+^ UAS-PICK1^A^-HA}* insertion used in the present study encodes the A-form and was generated earlier [20]. To produce the *P{w+UAS-PICK1A}* insertion, a cDNA encoding the PICK1 A-form, amplified from clone RE18409 (Drosophila Genomic Resource Center, Bloomington, IN), was inserted into the pCR-II-TOPO vector (Invitrogen, Carlsbad, CA) and subcloned as a *NotI-KpnI* fragment into the pUAST transformation vector [56]. Likewise, to produce the *P{w^+^ UAS-ICA69-HA}* insertion, full-length *CG10566* cDNA (Drosophila Genomic Resource Center, Bloomington, IN) and sequence encoding a C-terminal hemagglutinin tag was inserted into pCR-II-TOPO and subcloned as a *NotI*-*KpnI* fragment into pUAST. After sequencing, the pUAST plasmids were injected into embryos (Bestgene Inc., Chino Hills, CA). We used transformant ID 27281 (Vienna Drosophila RNAi Center, Vienna, Austria) to knock down expression of the *CG10566* gene that encodes the *Drosophila* homologue of mammalian ICA69. To increase knockdown efficiency, we expressed *dicer-2* using *P{w^+^ UAS-DCR-2.1}1* (Bloomington Stock Center, Bloomington, IN). To drive expression of the UAS transgenes, the following GAL4 insertions were used: *P{GawB}dimm^929^* [57], *P{Ilp2-GAL4.R}* [58], *P{Akh-GAL4.L}2* [59], and *P{GawB}elav^C155^*. When necessary, the various insertions were recombined onto chromosomes carrying either other insertions, *PICK1^1^*, or *PICK^2^*. Flies were reared on standard cornmeal medium (or on HSD for the metabolic experiments, see below) at 25°C at constant humidity, except during knockdown of ICA69 where the flies were kept at 28°C to maximize the effect of the RNAi construct. For determination of *Drosophila* body weights, flies were taken as progeny from vials seeded with 10 males and 10 females. One day after enclosure, 3–8 flies were placed in a preweighed Eppendorf tube and weighed. The weight of the tube was subtracted and the result divided by the number of flies.

### Mouse Genetics, Breeding, and Housing

The PICK1-deficient mice were generated as described [50]. The mice were backcrossed to C57BL/6 mice for three generations, and then bred to homozygosity using littermate male −/− and +/+ mice throughout the study. Mice backcrossed for more than 10 generations into C57BL/6 mice were used in selected experiments. In these mice we observed a similar reduction in body length, body weight, femur length, and GH and prolactin content in the pituitary as in the strain backcrossed for three generations. C57BL/6NTac mice (DIO mice) were purchased from Taconic (Hudson, NY) after they had been kept on a diet with 60% of the calories from fat for 10 wk from weaning at 6 wk. The mice were kept under the same diet upon arrival to University of Copenhagen and the next 2 wk. The lean control mice were likewise purchased from Taconic (Hudson, NY) and were at the same age and local breeding strain as the DIO mice. All animals were maintained and experiments conducted in accordance with institutional guidelines and approved by the Animal Experiments Inspectorate in Denmark.

### Measurement of Mouse Body Weight, Length, and Composition

Body weight was measured in mice at time points indicated in the figure legends. Body length between nose and tail root was measured in anesthetized mice at 16–18 wk of age [60]. Body composition was determined in live, unanesthetized animals by quantitative magnetic resonance imaging using EchoMRI 4-in-1 (Echo Medical Systems, Houston, TX). Femoral length was measured in mice at 39–41 wk of age. Mice were sacrificed and right femur was excised. After carefully removing all the adhering soft tissues, the length (cm) between the greater trochanter and medial condyle was measured. Indirect calorimetry was performed in a 16-chamber indirect calorimetry system (PhenoMaster: TSE Systems, Bad Homburg, Germany) to examine PICK1-deficient mice and WT mice at the age of 11–13 wk. Mice were individually housed and placed in the chambers for 8 d; the first 5 d was considered the acclimation phase, and data were analyzed only for the last 3 d. Oxygen consumption rate (VO_2_: ml/h/kg fat free mass), respiratory exchange ratio (RER), and food and intake were simultaneously measured for each mouse.

### Measurement of IGF-1, GH, and Other Pituitary Hormones

Mice at the age of 35–38 wk were used to determine plasma IGF-1 level. After an overnight fast, blood was collected from the orbital sinus and plasma was separated for measuring IGF-1 with ACTIVE Mouse/Rat IGF-1 RIA kit (Diagnostic Systems Laboratories, Inc.). Ghrelin-induced GH secretion was assessed in mice at the age of 7–9 wk. Fifteen minutes after anesthesia with pentobarbital (i.p. 50 mg/kg body weight), 10 µg of ghrelin (Polypeptide, Inc.) in 0.1 ml saline was injected i.p. Blood was sampled from orbital sinus before and 5 min after injection [26]. Plasma GH concentration was measured using Rat/Mouse GH ELISA KIT (Millipore) and plasma triglyceride (DT60II Model, Orthoclinical Diagnostics; Sollentuna, Sweden). To determine the total content of the protein level of pituitary-specific hormones, the pituitaries from 34-wk-old mice were dissected and lysed in RIPA Lysis buffer [1% Triton X-100, 150 mM NaCl, 10 mM Tris-HCl (pH 7.5), 1 mM EDTA, 1% NP-40, complete mini Protease Inhibitor Cocktail, ROCHE REF. 11836153001] and homogenizied. Three different pituitary hormones were measured using ELISA assay: GH (Millipore), prolactin and ACTH (Calbiotech), and TSH (Phoenix Peptides, Burlingame CA). Expression levels of GH and GH receptor mRNA were measured by quantitative real-time PCR in pituitaries taken from mice 35–38 wk old. Insulin receptor expression in liver samples was determined by Western blotting analysis on samples homogenized in the previously mentioned RIPA buffer. Protein extracts (20 µg/lane) were separated on 10% SDS-PAGE and electroblotted to PDVF membranes. Bands were quantified by densitometry (Alpha easy Innotech software). Equal loading was ensured with use of ß-actin antibody. The insulin receptor antibody was purchased from Santa Cruz Biotechnology, Inc. (Santa Cruz, CA).

### GH Treatment of PICK1-Deficient Mice

Recombinant human GH (rhGH) was a generous gift from Novo Nordisk (Norditropin Simplexx; Novo Nordisk, Denmark). Adult 17–21-wk-old PICK1-deficient mice were treated with GH (250 mg/20 g mouse) once daily for 3 wk. The mice were scanned in an echo MRI every week, and after the two first weeks of treatment, the mice were fasted for 16 h and challenged with an OGTT, and additionally 4 d later, the mice were exposed to an ITT after 2 h fasting. One experimental day and also 2 d before final termination by cervical dislocation, no GH was administrated. After termination the liver was dissected and frozen in liquid nitrogen in small pieces.

### Glucose Metabolism Measurements

OGTTs were carried out in 10–13-wk-old mice. The animals were fasted overnight for 16–18 h with free access to water. Glucose (1.5 g/kg body weight) was orally administered [43]. Blood glucose levels were monitored in the blood samples obtained from tail punctures using a handheld glucometer (Ascensia Contour Glucometer, Bayer) before and after glucose administration. At time points 0 and 15 min, blood was collected from the orbital sinus for measuring plasma insulin levels using Sensitive Insulin RIA KIT (Linco Research). Mice at age 27–29 wk were used for ITT. Food was removed 2 h before the test. Mice were i.p. injected with insulin at a dose of 0.75 U/kg body weight (Actrapid, Novo Nordisk, Denmark) [61]. Blood glucose levels were monitored as in the OGTT.

### Quantitative Real-Time PCR

Relative mRNA levels were measured by quantitative real-time PCR (RT-QPCR) using the Mx3000P from Stratagene and SYBR Premix Ex TaqTM (Takara) [62]. The relative levels of genes, from different samples, were compared by the ΔΔCt method, using the tyrosine 3-monooxygenase/tryptophan 5-monooxygenase activation protein, zeta polypeptide (YWHAZ), or hypoxanthine phosphoribosyltransferase 1 (HPRT1) as reference gene. In flies in which the internal control was *Rp49*, a gene encoding a ribosomal subunit was used. Before calculating the ΔΔCT value, primer efficiency was validated by standard curve measurements, and primers with more than 95% efficiency was used. A calibrator sample was included in each assay for normalization between runs. RNA was extracted with RNeasy Lipid Tissue Mini kit (Qiagen), and cDNA was synthesized by reverse transcription using the ImProm-IITM Reverse Transcriptase (Promega).

For RT-PCR in *Drosophila*, total RNA was purified from fly heads with Nucleospin RNA-II columns (Macherey-Nagel, Düren, Germany). For RT-PCR on liver samples, one steel bead together with 1 ml of QIAzol Lysis Reagent were added and mixed with a TissueRuptor. First strand synthesis was performed using Superscript III reverse transcriptase (Invitrogen). As internal control, the gene *Rp49* encoding a ribosomal subunit was used. RT-PCR was performed on a Mx3000P (Stratagene) using Platinum SYBR Green qPCR Supermix with ROX added as reference dye (Invitrogen).

### Immunohistochemistry

For immunostaining of *Drosophila* adult brain tissue, brains were dissected from female flies 24–48 h posteclosionin HL3 medium, fixed in PBS with 4% formaldehyde for 30 min on ice, and transferred to PBX (PBS+0,3% Triton-X100). After 6×10 min washes in PBX at RT, samples were blocked in PBX with 5% Normal Goat Serum for 30 min at RT and incubated with primary antibody overnight at 4°C. This was followed by 8×10-min washes in PBX at RT and secondary antibody incubation in PBX with 5% normal goat serum for 2 h at RT. Finally, the samples were washed in 8×10 min in PBX and 2×5 min in deionized water at RT and mounted in ProLong Gold antifade reagent (Invitrogen). Polyclonal rabbit anti-*Drosophila* PICK1 (SPY740, described in [20]) and polyclonal rabbit anti-*Drosophila* golgin-97 [63] were used in a concentration of 1∶300, polyclonal rat anti-dILP2 [64] was used in a concentration of 1∶800, and monoclonal rat anti-HA antibody (3F10, Roche) was used in a concentration of 200 pg/ml. As secondary antibodies, we used Alexa Fluor 488 goat anti-rabbit IgG (for PICK1 and golgin 97), Alexa Fluor 546 goat anti-rat IgG (for ICA69-HA), and Alexa Fluor 647 goat anti-rat IgG (for PICK1-HA and dILP2). For detection of dILP2, flies were kept on standard medium complemented with dried yeast.

For mouse immunohistochemistry, adult mice were sacrificed by decapitation, brains were rapidly removed, and pituitaries from PICK1 WT (*n* = 7) and PICK1-deficient mice (*n* = 7) were dissected out. Pituitaries were then rapidly frozen and kept at −80°C until further processing. Subsequently, pituitary sections were cut on a cryostat (15 µm) and mounted on superslide glasses (SuperFrost Plus, Menzel-Gläser, Braunschweig, Germany). Mounted pituitary sections were fixated in 4% paraformaldehyde (PFA) for 20 min and then rinsed in phosphate buffered saline (PBS) three times followed by preincubation for 30 min with 5% (v/v) goat serum in PBS containing 0.1% (v/v) Triton X-100. For PICK1 colocalization with hormones, sections were then incubated with mouse monoclonal PICK1 antibody (2G10, 1∶500), rabbit polyclonal GH antibody (1∶500, DAKO Cytomation A/S, Denmark), rabbit polyclonal ACTH antibody (1∶250, Abcam, UK), or rabbit prolactin antibody (1∶200, Abcam, UK). For PICK1 colocalization, studies with ICA69 mouse monoclonal PICK1 antibody (2G10, 1∶500) and rabbit polyclonal islet cell autoantigen (ICA-69) (1∶100, source, a kind gift from Massimo Pietropaolo, University of Michigan, Ann Arbor, MI, USA) [22] were incubated at 4°C overnight. On the second day, sections were rinsed in washing buffer (0.25% bovine serum albumin (BSA), 0.1% Triton X-100 in PBS) and incubated with secondary antibodies Alexa Fluor 488 goat anti-mouse IgG (for PICK1) and Alexa Fluor 568 goat anti-rabbit IgG (for ICA69 or GH) for 1 h (Molecular Probes, USA). This was followed by additional rinsing in washing buffer and PBS. Sections were finally coverslipped using Prolong Gold antifade reagent (Molecular Probes, Invitrogen).

### DNA Constructs

The plasmid encoding PICK1 N-terminally tagged with GFP was a kind gift from Dr. Kumlesh Dev (Trinity College, Dublin, Ireland). The constructs encoding YFP-PICK1, YFP-PICK1 BAR (Δ101), and YFP-PICK1 BAR (Δ101) 3KE in peYFP C1 (Clonetech, USA) were described previously [18]. The YFP-PICK1 BAR (Δ101) mutant V121E-L125E was generated by Quick-change (Stratagene) on YFP-PICK1 BAR and subsequently subcloned back into the peYFP C1 vector. The mycPICK1 construct in pCMV was a kind gift from Dr. Harvey T. McMahon (MRC Cambridge, UK) and was described previously [18]. GST PICK1 BAR (Δ101) in the pET41 vector was subcloned from previously described GST-PICK1 [65] and reintroduced in pET41 using MfeI and AvrII. The GST-PICK1 BAR (Δ101) mutant V121E-L125E was generated by Quick-change (Stratagene) on GST-PICK1 BAR and subsequently subcloned back into the pET41 vector [18]. HA-ICA69 in pcDNA5/FRT/TO was subcloned by PCR from a cDNA of the human sequence purchased from OriGene, introducing an N-terminal hemagluttinin (HA) tag. Cerulean-GalT was generated by Jennifer Lippincott-Schwartz and was purchased from Addgene (MA, USA). CgA-GFP [66] was kindly supplied by Dr. Laurent Taupenot. All constructs were verified by sequencing (MWG Operon, Germany).

### Cell Culture and Generation of Stable Cell Lines

GH1 cells were maintained in DMEM 1965 with Glutamax (L-alanyl-L-glutamine) containing 5% fetal calf serum and Pen/Strep at 37°C in a humidified 10% CO2 atmosphere [31]. ATT20 cells were maintained in DMEM 1965 supplied with 10% fetal calf serum and Pen/Strep at 37°C in a humidified 5% CO2 atmosphere. COS-7 cells were maintained in DMEM 1965 with Glutamax (L-alanyl-L-glutamine) containing 10% fetal calf serum and 0.01 mg/mL gentamicin (Invitrogen) at 37°C in a humidified 5% CO2 atmosphere. Cells were transfected using Lipofectamine 2000 (Invitrogen) and were used for experiments after 2 d. To generate a cell line with stable tetracycline-inducible expression of HA-ICA69, we used the FlpIn T-REx system and the Flp-In T-REx 293 cell line (Invitrogen). Cells (90% confluent) were transfected using Lipofectamine 2000 (Invitrogen) with a total of 3 µg of DNA in a 1∶9 ratio of the pcDNA5/FRT/TO with the HA-ICA69 insert, and pOG selection was induced using 15 µg/ml blasticidin and 150 µg/ml hygromycin. The stable HA-ICA69 expressing cells were maintained in DMEM 1965 containing 10% fetal calf serum at 37°C, and cells were selected using 15 µg/ml blasticidin and 100 µg/ml hygromycin (both from Invitrogen).

### Immunocytochemistry

For immunocytochemistry, cells were trypsinized and seeded on polyornithine-coated coverslips in six-well plates (150,000–300,000 cells/well depending on cell type). COS7 and Flp-In T-REx cells were transfected with Lipofectamine 2000 (Invitrogen) for 16 h in Optimem (Invitrogen) using a total of 1 µg DNA/well. In general, we transfected ∼80% of the COS7 cells and ∼40% of the Flp-In T-REx cells. For immunostaining, cells were washed in PBS (phosphate buffered saline, pH 7.4), fixed in 4% paraformaldehyde for 20 min, washed in PBS, and permeabilized by incubation for 30 min in PBS containing 5% goat serum and 0.2% saponin or 0.1% TX100 for COS7 cells. Primary antibodies including chicken anti-PICK1 (1∶500) (Novus Biologicals), rabbit ICA69 (1∶500), rabbit GH (1∶400) (DAKO Cytomation A/S, Denmark), mouse 58K (Abcam, UK), rabbit TGN38 (Sigma-Aldrich, USA), rabbit Giantin (Biosite, USA), rabbit GM130 (Sigma-Aldrich, USA), rabbit Ergic53 (Sigma-Aldrich, USA), mouse Syntaxin6 (3D10 Abcam, UK), mouse HA11 (Covance, USA), or rabbit LAMP1 (Abcam, UK) were added for 1 h followed by three washes and incubation with Alexa Fluor 488 goat anti-mouse IgG or Alexa Fluor 488 goat anti-rabbit IgG, together with Alexa Fluor 568 goat anti-chicken IgG (1∶500) (Molecular Probes, USA) for 30 min prior to mounting in Prolong Gold antifade reagent (Molecular Probes, USA). For labeling of the endocytic pathway (early and recycling endosomes), cells were fed Alexa Fluor 488–conjugated transferrin (Molecular Probes, USA) at 37°C for 1 h prior to PICK1 immunostaining as described above.

### RNAi-Mediated Knockdown in GH1 and ATT20 Cells

For GH1 and ATT20 RNAi studies, cells were used the BLOCK-iT Pol II miR RNAi Expression Vector Kit (Invitrogen, USA). Cells were transfected using Lipofectamine 2000 (Invitrogen, USA) with the pcDNA 6.2-GW vector, which has co-cistronic expression of miRNA with EmGFP (Emerald Green Fluorescent Protein), which permits visual selection of cells expressing the pre-miRNA. We used two pre-designed BLOCK-iT miR RNAi Select oligos against rat PICK1 and rat ICA69 (both PICK1 shRNAs but only one ICA69 shRNA worked in the mouse ATT20 cell line) as well as the negative control supplied. Two days after transfection, immunocytochemistry was performed as described above using mouse PICK1 2G10 (1∶500) (previously described) [20] together with either rabbit GH 1∶400 (DAKO Cytomation A/S, Denmark) or rabbit ICA69 1∶500. To allow for parallel visualization of EmGFP, the PICK1 antibody was visualized using Alexa Fluor 647 goat anti-mouse IgG and rabbit antibodies were visualized using Alexa Fluor 568 goat anti-rabbit IgG. For ATT20 cells, we used Alexa Fluor 568 goat anti-mouse IgG, and rabbit antibodies were visualized using Alexa Fluor 647 goat anti-rabbit IgG.

### Confocal Microscopy and Image Analysis

Visualization of mouse pituitary sections and cell lines was performed with a Zeiss LSM 510 inverted confocal laser-scanning microscope using an oil immersion numerical aperture 1.4 63× objective (Zeiss, Jena, Germany). The Alexa Fluor 488 dye, GFP, YFP, and EmGFP were excited with the 488 nm laser line from an argon–krypton laser, and the emitted light was detected using a 505–550 nm band pass filter. The Alexa Fluor 568 dye was excited at 543 nm with a helium–neon laser, and the emitted light was detected using a 560–615 nm band pass filter. For three color experiments, the Alexa Fluor 647 was excited at 633 nm with another helium-neon laser, and emitted light was detected using a 650 long pass filter. Channels were imaged separately. Resulting images were combined using IMAGEJ software (Rasband W. S., ImageJ, U.S. National Institutes of Health, Bethesda, MD, USA). *Drosophila* specimens were visualized using a Leica TCS SP2 confocal microscope (20× and 63× water immersion objectives), a Zeiss 510 upright confocal microscope with a C-Apochromat 63×/1.2 W Corr water immersion objective (Figure 7A and Figure S3G), or a Zeiss 710 upright confocal microscope using a Plan-Apochromat 63×/1.40 Oil DIC M27 oil immersion objective (Figure S3H). The Alexa Fluor 488 dye was excited with a 488 nm argon laser line, the Alexa Fluor 546 dye was excited with a 543 nm helium-neon laser, and the Alexa Fluor 647 dye was excited with a 633 nm helium-neon laser.

For quantification of PICK1, GH, and ICA69 immunoreactivity in GH1 and ATT20 cells, EmGFP expressing cells were outlined in the green channel, yielding separate regions of interest (ROIs), and the non-EmGFP expressing cells were grouped as a single ROI. Background levels were subsequently adjusted separately for each picture in the red and the blue channel before the total intensities of the ROIs were measured. Finally, the cumulated intensity of each EmGFP expressing ROI was compared to the average intensity of cells in the Non-EmGFP ROI within the same cell cluster. All image analysis was performed using IMAGEJ software (RasbandW. S., ImageJ, U.S. National Institutes of Health, Bethesda, MD, USA). Finally, the data were transferred to GraphPad Prism Statistical Software Version 4.0 for presentation and statistical analysis. The data did not show normal distribution according to the Shapiro-Wilk normality test. Hence, the Mann–Whitney Rank Sum Test was used for statistical analysis of data.

Quantification of co-localization was done using Van Steensel's cross-correlation function, which reports the Pearson cross-correlation as a function of the relative movement of the two channels with respect to each other. This was done using the JaCoP Plug-in for ImageJ [33] with the x-shift set to 20 pixels. Co-localization was quantified for 10–20 cells from three independent experiments. According to the cross-correlation function, completely co-localizing structures will show a cross-correlation function, peaking sharply at ΔX = 0 with a maximum value close to one, and mutually exclusive structures will tend to show no peak or even a dip at ΔX = 0. Partial co-localization will give rise to reduced maximum cross-correlation, as will noise. For adjacent and partially overlapping structures, the cross-correlation peak will become broader and/or shift away from a maximum at ΔX = 0 [33]. Quantification of punctate to total CgA-GFP signal in COS7 cells was done in ImageJ by thresholding the images and making a ROI excluding the coherent cluster of CgA-GFP containing the rest of the cell. The cumulated intensity outside the cluster was divided by the cumulated intensity for the entire cell. Finally, the data were transferred to GraphPad Prism Statistical Software Version 4.0 for presentation and statistical analysis.

### Time Lapse Confocal Microscopy

For time lapse confocal microscopy, GH1 cells were seeded in Labtek II eight-well chamber 24 h before co-transfection of YFP-PICK1, YFP-PICK1 BAR, or YFP-PICK1 BAR 3KE in the peYFP C1 vector (Clonetech, USA) with Cerulean-GalT in a 1∶3 ratio. Imaging was performed on the following day and was done at ∼35°C. Cerulean-GalT was exited with the 458 nm laser line and YFP with the 514 nm laser line from the argon laser and the emitted light directed through a 515 nm beam splitter and detected through 475–525 nm band pass and a 530 nm LP filter, respectively. We observed minimal cross-talk between the channels using these settings. We imaged an area of ∼24×24 µM (260×260 pixels) and switched between the two channels after every scanned line. Time lapses contained 100 frames with ∼3 s time resolution. Maximal intensity projections were carried out using ImageJ, and the presented images, time lapses, and supplemental movies were passed through the mean filter (radius 2 pixels) in ImageJ.

### Transmission Electron Microscopy

WT and PICK1−/− mice were fixed by vascular perfusion through the left ventricle of the heart with 2% glutaraldehyde in 0.05 M sodium phosphate buffer (pH 7.2) for 2 min. Following fixation, the pituitary was dissected from the brain, and the samples were rinsed three times in 0.15 M sodium cacodylate buffer (pH 7.2) and subsequently postfixed in 1% OsO4 in 0.12 M sodium cacodylate buffer (pH 7.2) for 2 h. The specimens were dehydrated in graded series of ethanol, transferred to propylene oxide, and embedded in Epon according to standard procedures. Ultrathin sections were cut with a Reichert-Jung Ultracut E microtome and collected on 200 mesh copper grids with Formvar supporting membranes. The sections were stained with uranyl acetate and lead citrate and examined with a Philips CM 100 transmission electron microscope operated at an accelerating voltage of 80 kV and equipped with a SIS MegaView II camera. Digital images were recorded with the analySIS software package.

### Giant Membrane Vesicle Deformation

Full-length PICK1 and PICK1 truncated at position 101 (PICK1 Δ101) [18] were expressed as GST (gluthathion-S-transferase) fusion proteins from pPET41 in *E. coli* BL21 DE3 pLysS cells and purified as described [49]. Cysteines in GST were fluorescently labeled with 100 µM Alexa 488 maleimide (Invitrogen) at 4°C for 1.5 h, washed three times with 10 ml buffer B+1 mM DTT, and eluted by addition of 10 mM glutathione. Purity and integrity of the protein were inspected by SDS–PAGE, and labeling efficiency was determined by fluorescence spectroscopy. Giant membrane vesicles were prepared from a lipid mixture containing 40% Phosphatidylcholine (DOPC), 32% Phosphatidylethanolamine (DOPE), 10% Phosphatidylserine (DOPS), 10% Cholesterol, 5% Phosphatidylinositol-4,5-bisphosphate (PtdIns(4,5)P_2_), 0.5% DOPE-biotin, and 0.5% DOPE-Atto633 (Attotec). All lipids were from Avanti polar lipids. The lipids were mixed from chloroform stocks and a thin lipid film was formed in a teflon cup upon solvent evaporation. The giant vesicles were generated by standard rehydration in TBS buffer (10 mM Tris, pH 7.4, and 95 mM NaCl) overnight at 37°C. Glass surfaces were passivated with a BSA:BSA-Biotin mixture and subsequently coated with streptavidin onto which the biotinylated vesicles tethered, as described previously [67]. The immobilized giant liposomes were incubated at room temperature with 0.8 µM–purified and Alexa Fluor 488–labeled GST-PICK1 Δ101 (BAR) or a mutant GST-PICK1 Δ101 (control) with impaired membrane-binding capacity (Val121Glu, Leu125Glu) (Madsen, Bhatia, Stamou, and Gether, unpublished observation). Subsequent microscopy was performed on a Leica TCS SP5 confocal fluorescence microscope, with AOBS/AOTF system allowing tunable wavelength detection intervals. The objective used was an oil immersion HCX PL APO with 100× magnification and numerical aperture 1.4. Vesicles and tubes containing DOPE-Atto633 dye were excited at 633 nm, detecting emission from 640 nm to 790 nm. The microscope was kept at a constant temperature of 22°C.

### Co-Immunoprecipitations

For co-immunoprecipitation of PICK1 and ICA69 from flies, heads were collected from female flies expressing *Drosophila* ICA69-HA and PICK1A under the control of the elav-GAL4 driver (*elav-GAL4/+; UAS-ICA69-HA/UAS-PICK1A*). The heads were homogenized on ice in modified RIPA buffer (20 mM Tris-HCl, 7.5 pH, 150 mM NaCl, 10 mM MgCl_2_, 0.5% NP-40, 1 mM PMSF, 1 Complete Protease Inhibitor Cocktail tablet (Roche) per 25 ml buffer). The resulting homogenate was passed through a 25 gauge needle seven times and centrifuged at 20,800× *g* at 4°C until the supernatant was clear. The concentration of the supernatant was subsequently adjusted to 1 mg/ml protein. One ml of lysate per binding reaction was cleared with 15 µl Protein-G Agarose beads (Roche) for 1 h. Beads were removed by centrifugation at 2,700× *g* for 5 min at 4°C, and 4 µg/ml rat anti-HA (3F10, Roche) was added to the supernatant. Immune complexes were allowed to form for 12 h before the addition of 15 µl Protein-G Agarose beads, followed by an additional 4 h of incubation. All incubations were done at 4°C under gentle agitation. Beads were then pelleted by centrifugation at 2,700× *g* for 5 min at 4°C and washed 4 times with 1 ml modified RIPA buffer. The bound material was eluted by boiling for 5 min in loading buffer before analysis by SDS-PAGE and immunoblotting with mouse anti-HA (16B12, Covance) to detect immunoprecipitated ICA69-HA and rabbit anti-*Drosophila* PICK1 (SPY740) [9] to detect coimmunoprecipitated endogenous PICK1, respectively.

For co-immunoprecipitation in GH1 cells, cells were grown to confluence and lysed for 30 min at 4°C in lysis buffer (PBS+1% Triton X-100) supplemented with Complete protease Inhibitor Cocktail tablet (Roche, Mannheim, Germany). Protein G agarose beads (Roche, Mannheim, Germany) were preincubated 1 h with monoclonal 2G10 mouse anti-PICK1 antibody, and the cell lysate was cleared by spinning 30 min at 16,000 rpm at 4°C in a tabletop centrifuge. The resulting supernatant was transferred to the antibody-agarose bead mix, and the sample was incubated for 1 h at 4°C under constant rotation. Lysates incubated with agarose beads without antibody were included as control. After incubation, samples were washed once in lysis buffer, twice in PBS with 1% Triton X-100 and 500 mM NaCl, and three times in PBS. After washing, protein was eluted from the beads by adding SDS-PAGE loading buffer. Samples were analyzed by SDS-PAGE and immunoblotted with polyclonal guinea pig anti-PICK1 and rabbit anti-ICA69 to detect immunoprecipitated PICK1 and coimmunoprecipitated ICA69, respectively.

### HSD Model in *Drosophila*

To establish the HSD model [38]–[40], WT flies were allowed to deposit eggs on an apple juice plate for 6 h. On the following day, first instar larvae hatched from the eggs were transferred to vials either containing food with a standard amount of sucrose or five times that amount (HSD). The food contained (g/L): agar, 10; yeast, 34; corn meal, 82.5, sucrose, 60 (for standard food) or 300 (for HSD) and also included mold inhibitors (ml/L): Nipagin in ethanol, 15; propionic acid, 2. Quantitative RT-PCR was performed according to methods described above on mRNA isolated from pooled heads of fully developed adult female flies reared on standard food (*n* = 9 samples), or on HSD (*n* = 7).

### Statistical Analysis

Statistical analysis was performed using GraphPad Prism Statistical Software Version 4.0. Two-tailed Student's *t* test was used for comparison between two groups. One-way ANOVA followed by Bonferroni post hoc tests was used to analyze body weight and composition in mice. Two-way repeated measures ANOVA followed by Bonferroni post hoc tests were used for the analysis of data of ghrelin-induced GH secretion, OGTT, insulin level during OGTT, and ITT. All data are presented as mean ± SE. We considered *p*<0.05 significant. Other statistical procedures are described in the relevant sections.

## Supporting Information

Figure S1 Body composition of PICK1-deficient mice and measurement in PICK1-deficient mice of water intake, food intake, energy expenditure, and respiratory exchange rate by indirect calorimetry. (A and B) Lean mass and fat mass were determined in live, unanesthetized mice at 15–17 wk of age by quantitative magnetic resonance scanning. Data are given as total amount of fat and lean body mass and presented as means ± SE (*n* = 7–10). ***p<0.001, compared to WT mice; ^##^ *p*<0.01, compared to KO mice. (C to H) Measurement of water intake, food intake, energy expenditure, and respiratory exchange rate was performed in cages optimized for indirect calorimetry with individual scales for food and water. Indirect calorimetry in PICK1-deficient and WT mice were done at the age of 11–13 wk with mice placed in calorimetry cages for 8 d. (C) Average 12 h water intake and (D) food intake in the light and dark period for WT (+/+)(white column) and GPR39-deficient (−/−) mice (black column). (E) Average 12 h oxygen consumption rate (VO_2_: ml/h/kg fat free mass) and (F) respiratory exchange rate in the light and dark period for WT (+/+)(white column) and GPR39-deficient (−/−) mice (black column). (G) Oxygen consumption rate (VO_2_: ml/h/kg fat free mass) and (H) respiratory exchange rate in the dark and light period for WT (+/+) (black line) and PICK1-deficient (−/−) (grey line). Data were analyzed by one-way ANOVA followed by Bonferroni post hoc test. Data are shown as means ± SE, *n* = 7.
(EPS)

Figure S2 Protein level, total protein of ACTH, prolactin (in ng/µg), and TSH (in uU/ug) measured by ELISA in pituitary extracts taken from 34-wk-old WT (+/+) and PICK1-deficient (−/−) mice. Data are expressed as means ± SE (*n* = 4). *p<0.05 compared to WT mice.
(EPS)

Figure S3 Immunohistochemical localization of PICK1 to ACTH and prolactin-producing cells in mice pituitary. Confocal images of 15 µm slices from the pituitary of WT (+/+) mice, immunostained for PICK1 (green) and ACTH (red) (top), and PICK1 (green) and prolactin (red) (bottom). Insets show cells expressing both PICK1 and ACTH (top) or prolactin (bottom). Scale bar, 10 µm. Pictures are representative of stainings of three pituitaries.
(EPS)

Figure S4 shRNA-mediated knockdown of PICK1 reduces ACTH immunosignal in ATT20 cells. (A) PICK1 is expressed in ACTH-producing ATT20 cells, and PICK1 knockdown reduces apparent ACTH content. Confocal images of ATT20 cells immunostained for PICK1 (red) and ACTH (red). Cells transfected with shRNA against PICK1 are identified by obligate co-expression of green fluorescent protein (EmGFP) (green) and are outlined in white. Scale bar, 10 µm. (B and C) Quantification of PICK1 immunosignal (B) and ACTH immunosignal (C) in GFP-positive cells transfected with either of two different shRNAs against PICK1 (52 and 53). Data are percentage of signal in surrounding nontransfected cells (mean ± SE) for control shRNA (shControl) (*n* = 25), shPICK1 (52) (*n* = 46), and shPICK1 (53) (*n* = 24). ***p<0.001, **p<0.01, compared to shControl, Mann–Whitney rank sum test.
(EPS)

Figure S5 Co-localization of PICK1 with vesicular markers in GH1 cells. Confocal images of GH1 cells immunostained for PICK1 and indicated endocytic pathway markers. Left panels show signal from immunolabeled PICK1 (Alexa Fluor 568 signal), middle panels signal from immunolabeled endocytic marker (Alexa Fluor 488 signal), and right panels overlay of the two channels. (A) Transferrin (early and recycling endosomes). (B) LAMP1 (lysozomes). (C) Ergic 53. Insets highlight lack of co-locolization of PICK1 with transferrin and LAMP1 and partial colocalization with Ergic 53. Pictures are representative of three to six independent experiments. (D) Quantification of the PICK1 colocalization with tranferrin, LAMP1, and Ergic 53 using Van Steensel's cross-correlation function, which report the Pearson cross-correlation as a function of the relative movement of the two channels with respect to each other. Neither transferrin nor LAMP1 co-localize with PICK1, whereas the sharp peak close to Δx = 0 indicates partial but specific co-localization for Ergic 53. Co-localization was quantified for 10–20 cells from three independent experiments, and data are means ± SE.
(EPS)

Figure S6 Confocal images of GH1 cells immunostained for PICK1 and indicated markers and of HA-tagged PICK1A and indicated markers in the adult *Drosophilia* brain. (A and B) Confocal laser scanning micrographs of GH1 cells immunostained for PICK1 and indicated Golgi markers. Left panels show signal from immunolabeled PICK1 (Alexa Fluor 568 signal), middle panels signal from immunolabeled Golgi marker (Alexa Fluor 488 signal), and right panels overlay of the two channels. (A) GM130 (cis-Golgi) and (B) 58K (entire Golgi). Insets highlight adjacent localization rather than co-localization of PICK1 with Golgi markers. Pictures are representative of three to six independent experiments. (C) Immunostainings for HA-tagged PICK1A (red) and golgin 97 (green) in a single cell in lateral protocerebrum of the adult *Drosophilia* brain. PICK1A-HA was expressed using the c929-GAL4 driver (*c929-GAL4 PICK^1^/UAS-PICK1A-HA PICK^2^*). PICK1A-HA is seen as clusters that are primarily next to the staining for the trans-Golgi marker, golgin-97, rather directly overlapping. (D) Immunostainings for endogenous PICK1 (green) and dILP2 (red) in median neurosecretory cells of the pars intercerebralis in the adult *Drosophila* brain. The PICK1 staining shows partial overlap with DILP2 staining but mostly clusters around zones of high dILP2 immunoreactivity. Scale bars are 5 µm in panels and 1 µm in insets, respectively. (E and F) Punctate co-localization of PICK1, syntaxin 6, and GH in GH1 cells. (E) Confocal laser scanning micrographs of GH1 cells with the first image (from left) showing signal from immunolabeled PICK1 (Alexa Fluor 568 signal, red), second image signal from syntaxin 6 (Alexa Fluor 488 signal, green), third image from GH (Alexa Fluor 647 signal, blue), and fourth panel overlay of the three channels. The PICK1 and syntaxin 6 signals are oversaturated in the perinuclear region to enable visualization of the weaker punctuate expression together with the exclusively punctate GH signal (blue). (F) Representative intensity profiles from (E) indicated by i, ii, and iii through numerous vesicles demonstrating several incidents of co-localization of the immunosignals from PICK1 (red), syntaxin6 (green), and GH (blue).
(EPS)

Figure S7 Punctate localization adjacent to the Golgi compartment of GFP-PICK1 BAR in GH1 cells. PICK1 BAR tagged N-terminally with GFP (GFP-PICK1 BAR) (left) or the mutants GFP-PICK1 BAR V121E-L125E (middle) and GFP-PICK1 BAR 3KE (right) (all shown in yellow) were expressed in GH1 cells together with the Golgi marker GalT, β1,4-galactosyltransferase fused to cerulean fluorescent protein (GalT-cerulean) (shown in blue). Confocal imaging showed a punctate localization of YFP-PICK1 BAR with many punctae lining the GalT-cerulean visualized Golgi compartment. PICK1 BAR V121E-L125E showed a similar punctate localization, whereas GFP-PICK1 BAR 3KE localized diffusely in the cytoplasm. The erased white areas correspond to the nuclei of the cells.
(EPS)

Figure S8 ShRNA-mediated knockdown of PICK1 reduced ICA69 expression in ATT20 cells and vice versa. (A) Confocal images of ATT20 cells immunostained for PICK1 and ICA69. Cells were transfected with shRNA against PICK1 and identified by obligate co-expression of EmGFP (cells lined in white). First image (from left) shows EmGFP signal, second image signal from PICK1 (Alexa Fluor 568 signal), third image signal from immunolabeled ICA69 (Alexa Fluor 647 signal), and fourth panel image overlay of the three channels. Pictures are representative of multiple cells imaged on three independent occasions. Scale bar, 10 µm. (B) Quantification of PICK1 expression and (C) ICA69 expression in GFP-positive cells transfected with either of two different shRNAs (52 and 53) against PICK1. Data are presented as percent of expression in surrounding nontransfected cells (means ± SE) for control shRNA (shControl) (*n* = 36), shICA69 (24) (*n* = 55), and shPICK1 (25) (*n* = 24). (D) Confocal images of ATT20 cells immunostained for PICK1 and ICA69. Cells were transfected with shRNA against ICA69 and identified by obligate co-expression of EmGFP (cells lined in white). First image (from left) shows EmGFP signal, second image signal from PICK1 (Alexa Fluor 568 signal), third image signal from immunolabeled ICA69 (Alexa Fluor 647 signal), and fourth panel image overlay of the three channels. Pictures are representative of multiple cells imaged on three independent occasions. Scale bar, 10 µm. (E) Quantification of ICA69 expression and (F) PICK1 expression in GFP positive cells transfected with either of two different shRNAs (24 and 25) against ICA69 (sh25 not quantified for PICK1 since it does not knock down ICA69 in ATT20 cells). Data are presented as percent of expression in surrounding nontransfected cells (means ± SE) for control shRNA (shControl) (*n* = 36), shICA69 (24) (*n* = 26), and shPICK1 (25) (*n* = 29). *** indicate that medians are significantly different from the control with *p*<0.001 as determined by Mann–Whitney rank sum test (data do not show normal distribution).
(EPS)

Figure S9 ShRNA-mediated knockdown of ICA69 reduces PICK1 expression in GH1 and ATT20 cells. (A) Confocal laser scanning micrographs of GH1 cells immunostained for ICA69 and PICK1. Cells were transfected with shRNA against ICA69 and identified by obligate coexpression of EmGFP (cells lined in white). First image (from left) shows signal from immunolabeled ICA69 (Alexa Fluor 568 signal), second image signal from PICK1 (Alexa Fluor 647 signal), third image EmGFP signal, and fourth panel image overlay of the three channels. Pictures are representative of multiple cells imaged on three independent occasions. Scale bar, 10 µm. (B) Quantification of ICA69 expression and (C) PICK1 expression in GFP-positive cells transfected with either of two different shRNAs (24 and 25) against ICA69. Data are presented as percent of expression in surrounding nontransfected cells (means ± SE) for control shRNA (shControl) (*n* = 49), shICA69 (24) (*n* = 72), and shPICK1 (25) (*n* = 65). *** indicate that medians are significantly different from the control with *p*<0.001 as determined by Mann–Whitney rank sum test (data do not show normal distribution).
(EPS)

Figure S10 PICK1 stabilizes HA-ICA69 expression in Flp-In T-Rex 293 cells. Flp-In T-Rex 293 cells stably expressing HA-tagged ICA69 (HA-ICA69) in a tetracycline-dependent manner (Flp-In T-Rex 293 HA-ICA69 cells) were transfected with pcDNA3 or mycPICK1 before induction of the cells with tetracycline for 24 h. Cell lysates were subsequently analyzed by Western blotting. (Left) The gel shows three independent experiments with HA-ICA69 on top and Actin at bottom as loading control. (Right) Quantification of the HA-ICA69 signal normalized to Actin. Data are means ± SE, ***p<0.001 in Student's *t* test.
(EPS)

Movie S1 Time lapse series of YFP-PICK1 BAR and GalT-Cerulean showing highly mobile punctate structures of YFP-PICK1 BAR concentrated at the lining of the Golgi. In addition, several punctuate structures appear and disappear during the time lapse series. The movie is 100 frames with 3 s intervals (total time ∼5 min). The size is ∼24×24 µm.
(MOV)

Movie S2 Time lapse series of YFP-PICK1 BAR V121E-L125E and GalT-Cerulean showing mainly stable punctate structures of YFP-PICK1 BAR V121E-L125E both at the lining of the Golgi and elsewhere in the cell. Few punctuate structures appear and disappear during the time lapse series. The movie is 100 frames with 3 s intervals (total time ∼5 min). The size is ∼24×24 µm.
(MOV)

## Abbreviations

BAR: Bin/amphiphysin/Rvs
BFA: brefeldin A
CgA: chromogranin A
DCVs: dense core vesicles
dILPs: *Drosophila* insulin-like peptides
DIO: diet-induced obesity
GalT: 1,4-galactosyltransferase
GFP: green fluorescent protein
GH: growth hormone
HA: hemagglutinin
HSD: high-sugar diet
ICA69: islet cell autoantigen 69
IGF-1: insulin-like growth factor-1
ITT: insulin tolerance test
OGTT: oral glucose tolerance test
PICK1: protein interacting with C kinase 1
PDZ: PSD-95/Discs-large/ZO-1
shRNA: small hairpin RNA
TGN: trans-Golgi network
WT: wild type
